# Construction of Full‐Spectrum‐Response Bi_3_O_4_Br:Er^3+^@Bi_2_O_3‐_ *_x_* S‐Scheme Heterojunction With [Bi─O] Tetrahedral Sharing by Integrated Upconversion and Photothermal Effect Toward Optimized Photocatalytic Performance

**DOI:** 10.1002/advs.202412214

**Published:** 2025-01-02

**Authors:** Zhifeng Li, Liang Xu, Zhaoyi Yin, Junhao Ma, Xiaoyi Dong, Shangyong Wang, Zhiguo Song, Jianbei Qiu, Yongjin Li

**Affiliations:** ^1^ Faculty of Materials Science and Engineering Kunming University of Science and Technology Kunming 650093 P. R. China

**Keywords:** [Bi─O] tetrahedral sharing, Bi_3_O_4_Br:Er^3+^@Bi_2_O_3‐x_, full‐spectrum‐response, photothermal effect, S‐scheme heterojunctions

## Abstract

Designing and optimizing photocatalysts to maximize the use of sunlight and achieve fast charge transport remains a goal of photocatalysis technology. Herein, a full‐spectrum‐response Bi_3_O_4_Br:Er^3+^@Bi_2_O_3‐_ *_x_* core–shell S‐scheme heterojunction is designed with [Bi─O] tetrahedral sharing using upconversion (UC) functionality, photothermal effects, and interfacial engineering. The UC function of Er^3+^ and plasmon resonance effect of Bi_2_O_3‐_ *_x_* greatly improves the utilization of sunlight. The equivalent layer structure of Bi_3_O_4_Br and Bi_2_O_3‐_ *_x_* facilitates the construction of high‐quality S‐scheme heterojunction interfaces with close atomic‐level contact obtained from the [Bi─O] tetrahedral sharing and the resulting Bi_3_O_4_Br:Er^3+^@Bi_2_O_3‐_ *_x_* core–shell morphology, enabled efficient charge transfer. Furthermore, localized temperature increase, induced by photothermal effects, enhanced the chemical reaction kinetics. Benefiting from the distinctive construction, the Bi_3_O_4_Br:Er^3+^@Bi_2_O_3‐_ *_x_* heterojunctions exhibit excellent performance in the photocatalytic degradation of bisphenol A that is 2.40 times and 4.98 times greater than that of Bi_3_O_4_Br:Er^3+^ alone under full‐spectrum light irradiation and near‐infrared light irradiation, respectively. This work offers an innovative perspective for the design and fabrication of full‐spectrum‐response S‐scheme heterojunction photocatalysts with efficient solar energy utilization based on high quality interfaces, UC functionality, and the photothermal effect.

## Graphical abstract

Bi_3_O_4_Br:Er^3+^@Bi_2_O_3‐_ *_x_* core–shell S‐scheme heterojunction with [Bi─O] tetrahedral sharing is designed and prepared, that integrating upconversion functionality, photothermal effect, and interfacial engineering. Benefiting from the distinctive construction, the Bi_3_O_4_Br:Er^3+^@Bi_2_O_3‐_ *_x_* heterojunction exhibits excellent photocatalytic performance.

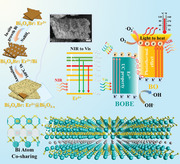

## 1 Introduction

Photocatalytic technology, which utilizes solar energy to create renewable and clean energy while degrading pollutants in the environment, has developed rapidly in recent years as one of the most viable solutions to the increasingly serious problems of shortages in conventional energy supplies and environmental pollution.^[^ ^1^ ^]^ For example, bismuth‐based semiconductor materials have shown great promise within the field of photocatalysis due to their unique crystal structures, complex atomic coordination, and diverse compositions.^[^ ^2^ ^]^ Among these, non‐stoichiometric Bi_3_O_4_Br has generated considerable interest due to its excellent photocatalytic activity in CO_2_ reduction,^[^ ^3^ ^]^ nitrogen fixation,^[^ ^4^ ^]^ and organic pollutant degradation^[^ ^5^ ^]^ owing to the wide internal space provided by its unique layered crystal structure, which helps to polarize the relevant atoms and orbitals, and thereby promotes the effective separation of electrons and holes.^[^ ^6^ ^]^ However, the full practical application of existing photocatalytic technology remains limited by its low conversion efficiency to solar energy.^[^ ^7^ ^]^ An important cause of this low efficiency is the narrow response of photocatalysts to the solar spectrum, where most photocatalysts currently respond only to ultraviolet (UV) and visible (Vis) light, while nearly 53% of the near‐infrared (NIR) component of the solar spectrum is not properly utilized.^[^ ^8^ ^]^ Therefore, increasing the response of photocatalysts to NIR light holds significant promise for advancing the practical applications of photocatalytic technology.

One possible means of addressing this issue is to take advantage of the well‐known optical property of upconversion (UC) materials for converting NIR light into UV or Vis light.^[^ ^9^ ^]^ In this regard, lanthanide‐doped UC luminescent materials are particularly attractive because a well‐designed doping structure enables efficient conversion for NIR light at a wavelength of 980 nm, which represents a substantial portion of the solar spectrum.^[^ ^10^ ^]^ Hence, a number of lanthanide‐doped UC materials have been applied to increase the response of photocatalysts to NIR light, such as ZIF‐67/Ag NPs/NaYF_4_:Yb,Er,^[^ ^11^ ^]^ NaYF_4_:Yb/Tm@Ag_3_PO4/Ag@g‐C_3_N_4_,^[^ ^12^ ^]^ and NaYF_4_:Yb,Gd,Tm@Bi_2_WO_6_.^[^ ^13^ ^]^ In terms of lanthanide‐doped bismuth‐based semiconductor photocatalysts, a number of materials, such as BiOBr:Yb^3+^/Er^3+^,^[^ ^14^ ^]^ Bi_4_O_5_I_2_:Er^3+^/Yb^3+^,^[^ ^15^ ^]^ and Bi_4_O_5_I_2_/BiOBr:Yb^3+^, Er^3+^,^[^ ^16^ ^]^ have exhibited excellent photocatalytic activity under NIR light irradiation for the degradation of organic pollutants compared to their undoped counterparts.

Beyond the above‐discussed issue of full solar energy utilization, optimizing the efficiency of separating photogenerated charge carriers in photocatalysts is also a central challenge.^[^ ^17^ ^]^ In this regard, the construction of heterojunction structures has been shown to reduce the recombination of photogenerated charge carriers significantly.^[^ ^18^ ^]^ Recent work has proposed an innovative S‐scheme heterojunction structure that cleverly combines a reduction photocatalyst (RP), which relies on photogenerated electrons in a photocatalysis process generally applied for the production of fuels, and an oxidation photocatalyst (OP), which relies on photogenerated holes in a photocatalysis process generally applied for the degradation of environmental pollutants.^[^ ^19^ ^]^ In this heterojunction, the electrons and holes respectively generated by photoexcitation in the RP and OP follow a unique stepwise transfer path at the interface between the two catalysts. The core of the S‐scheme heterojunction mechanism lies in the clever arrangement of the energy band structure, which introduces an internal electric field (IEF) at the RP/OP interface.^[^ ^20^ ^]^ Specifically, the IEF acts as a driving force to induce the combination of electrons, which have a weaker reduction ability in the OP, with holes, which have a weaker oxidation ability in the RP.^[^ ^21^ ^]^ This process produces a stronger redox capacity of the retained electrons and holes in each phase of the catalyst. In addition, this process not only substantially improves the efficiency of charge carrier separation, but also endows the photocatalytic system with more significant photoreduction and photodegradation capabilities. Hence, the selection of suitable narrow‐band semiconductor materials is particularly crucial for optimizing the light‐absorbing capacity and charge carrier separation efficiency of S‐scheme heterojunctions.^[^ ^22^ ^]^

In addition to the energy band structure of S‐scheme heterojunctions, the quality of atomic contacts at the RP/OP interface is another critical factor affecting the performance of S‐scheme heterojunctions. Recently, the close atomic‐level contact obtained at the interface of heterojunctions that include shared elements (i.e., atom co‐sharing heterojunctions), such as metallic Bi and a Bi‐based semiconductor like Bi_4_O_5_Br_2_ (i.e., a Bi/Bi_4_O_5_Br_2_ heterojunction), have been demonstrated to achieve improved photocatalytic performance by promoting efficient interfacial charge transfer and robust interface interactions.^[^ ^23^ ^]^ Similarly, the shared interface formed by the Sn atoms in Cs_2_SnI_6_/SnS_2_ heterojunctions have been demonstrated to extend the charge carrier lifetime.^[^ ^24^ ^]^ Meanwhile, the full photoreduction of Cr(VI) to Cr(III) was realized within 10 min for photocatalysts involving BiOBr/Bi_2_S_3_ S‐scheme heterojunctions due to the promotion of interfacial charge transfer via [Bi‐0] tetrahedral sharing.^[^ ^25^ ^]^ Finally, the incorporation of morphological design features, such as core–shell structures, can further benefit the photocatalytic activity of S‐scheme heterojunction photocatalysts by increasing the interfacial contact area and the concentration of exposed active sites.^[^ ^26^ ^]^

In addition to the great promise shown by bismuth‐based semiconductor materials within the field of photocatalysis, the localized surface plasmon resonance (LSPR) effect induced in Bi_2_O_3‐_ *_x_* by its variable concentration *x* of oxygen vacancies (OVs) makes it a special plasma material with significant advantages over conventional plasma metals, such as gold (Au), silver (Ag), and platinum (Pt), in terms of low cost, good safety performance, and its diverse physical properties.^[^ ^27^ ^]^ In addition, Bi_2_O_3‐_ *_x_* has a broad LSPR spectral range spanning 600 to 1400 nm. More importantly, the optical properties of Bi_2_O_3‐_ *_x_*, including the photoresponsive region of the LSPR and its intensity, can be manipulated by finely tuning the concentration of OVs.^[^ ^28^ ^]^ Of further interest is that both Bi_3_O_4_Br and Bi_2_O_3‐_ *_x_* contain a [Bi_2_O_2_]^2+^ layer structure. This property greatly facilitates the growth of Bi_2_O_3‐_ *_x_* on the surface of Bi_3_O_4_Br in situ, which enables the construction of high‐quality heterojunction interfaces with close atomic‐level contact obtained from the [Bi─O] tetrahedral sharing. In addition, increases in the temperature of the reaction system arising from the strong photothermal conversion ability of Bi_2_O_3‐_ *_x_* arising from plasma resonance would further enhance the photocatalytic performance of Bi_3_O_4_Br/Bi_2_O_3‐_ *_x_* heterojunctions by promoting the mobility of photogenerated carriers and the redox reaction rate through the photothermal effect.^[^ ^29^ ^]^ Nonetheless, to the best of authors' knowledge, efforts to enhance the full‐spectrum responsiveness of bismuth‐based materials via the implementation of lanthanide‐doped Bi_3_O_4_Br:Er^3+^ and Bi_2_O_3‐_ *_x_* heterojunctions remain poorly explored. Moreover, the benefits of employing core–shell morphologies for enhancing the photocatalysis process involving bismuth‐based UC materials have not been systematically investigated.

The present work addresses the above‐discussed limitations in past work by designing particulate Bi_3_O_4_Br:Er^3+^/Bi_2_O_3‐_ *_x_* S‐scheme heterojunctions with Er^3+^ doping and [Bi─O] tetrahedral sharing in the form of a Bi_3_O_4_Br:Er^3+^@Bi_2_O_3‐_ *_x_* core–shell morphology. In addition, experimental Bi_3_O_4_Br:Er^3+^@Bi_2_O_3‐_ *_x_* samples are fabricated via a solvothermal‐calcination tandem synthesis strategy. The results of experiments demonstrate that the as‐prepared Bi_3_O_4_Br:Er^3+^@Bi_2_O_3‐_ *_x_* materials provide excellent photocatalytic activity in bisphenol A (BPA) degradation under full‐spectrum light irradiation. A combination of experimental and density functional theory (DFT) calculations confirm that the charge transfer mechanism of the designed material is representative of an S‐scheme heterojunction. Moreover, the results of photothermal experiments confirm that the Bi_3_O_4_Br:Er^3+^@Bi_2_O_3‐_ *_x_* heterojunction material promotes photocatalytic reactions via the photothermal effect. Finally, the experimental results are applied to propose a possible photocatalytic enhancement mechanism for the proposed heterojunctions.

## 2 Results and Discussion

### 2.1 Morphology and Structural Characterization

**Figure 1:**
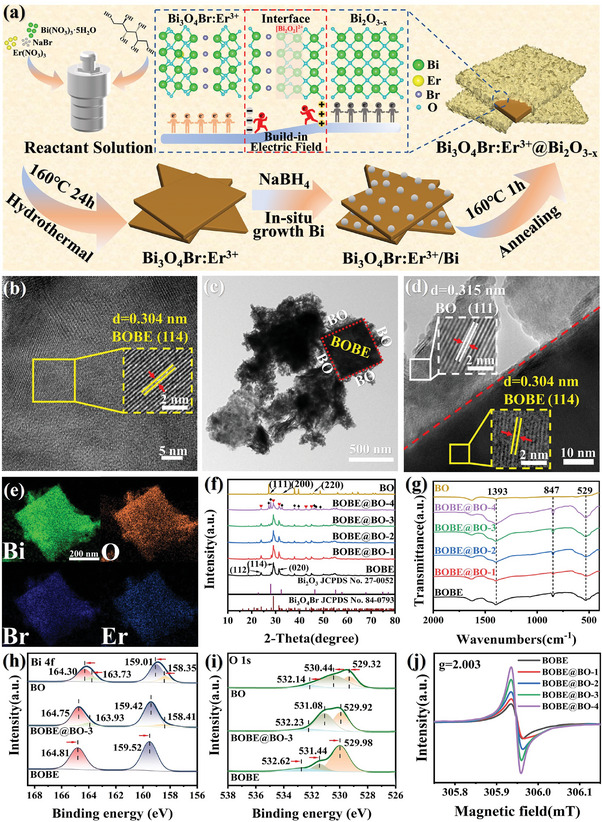
a) Schematic illustrations of the synthetic process and crystal structure; b) HRTEM of BOBE; c) TEM, d) HRTEM and e) EDS elemental mapping of BOBE@BO‐3; f) XRD and g) FT‐IR spectra of BOBE, BO and BOBE@BO; XPS spectra of h) Bi 4f and i) O 1s spectra of BOBE, BOBE@BO‐3 and BO; j) EPR spectra of BOBE@BO heterojunctions.

The Bi_3_O_4_Br:Er^3+^@Bi_2_O_3‐_ *_x_* heterojunctions were grown in situ using the solvothermal‐calcination tandem synthesis process illustrated in **Figure** **1**a. The detailed synthesis process is described in the Experimental section and Supporting information. Briefly, Bi_3_O_4_Br:Er^3+^ nanoplates (BOBE) were initially prepared using the solvothermal method. These nanoplates were then added to different concentrations of NaBH_4_ solutions (5, 10, 15, and 20 mM), and Bi_3_O_4_Br:Er^3+^/Bi was obtained whose characterization is shown in Figures S1 and S2 (Supporting Information). subsequently subjected to low‐temperature annealing. The Bi_3_O_4_Br:Er^3+^@Bi_2_O_3‐_ *_x_* heterojunctions are denoted herein as BOBE@BO‐1, BOBE@BO‐2, BOBE@BO‐3, BOBE@BO‐4 according to the 5, 10, 15, and 20 mM concentration of added NaBH_4_ solution, respectively. For comparison, pure Bi_2_O_3‐_ *_x_* (BO) samples were synthesized. As can be seen in Figure 1a, the BOBE atomic structure consists of alternating [Bi_2_O_2_]^2+^ and [Br]^−^ layers.^[^ ^30^ ^]^ The illustration further shows that the Bi─O tetrahedral structure of the [Bi_2_O_2_]^2+^ layer in BOBE is similar to that of BO,^[^ ^31^ ^]^ which facilitates the growth of BO in situ on BOBE with [Bi─O] tetrahedral sharing. The strong interfacial interactions in this high‐quality interface can be expected to provide a highway for charge carrier transfer, and thus reducing carrier complexation and, while also enhancing the structural stability of the heterostructure photocatalysts.^[^ ^25^ ^]^

The surface morphologies and microstructure of the synthesized samples were explored via scanning electron microscopy (SEM) and transmission electron microscopy (TEM). A 2D nanoplate shape of BOBE can be discerned based on the SEM image in Figure S3a (Supporting Information), which reveals a particle size of 200–500 nm. This 2D morphology is confirmed by the corresponding TEM images in Figure S3b (Supporting Information). The lattice fringes observed in the high‐resolution TEM (HRTEM) image presented in Figure 1b have a spacing of 0.304 nm, which can be ascribed to the (114) crystal plane of Bi_3_O_4_Br. The SEM image presented in SI for the BOBE@BO‐1 sample (Figure S3c, Supporting Information) indicates that the morphology is similar to that of BOBE, but the small nanosheets are attached to the surface with tight contact. We can infer from the reported images that the 2D morphology of BOBE provides a large surface area and good substrate for the homogeneous nucleation growth of BO nanosheets. The SEM images in Figure S3d–f (Supporting Information) clearly demonstrate that the BOBE nanoplate surfaces become covered by an increasing density of interlaced BO nanosheets with increasing BO content, which resulted in the formation of a core–shell structure. The growth of a shell of BO nanosheets around a BOBE core is further illustrated by the TEM image of BOBE@BO‐3 presented in Figure 1c. The HRTEM image of BOBE@BO‐4 presented in Figure 1d exhibits distinct lattice fringes with facet spacing values of ≈ 0.304 and 0.318 nm, which respectively correspond to the (114) plane of Bi_3_O_4_Br and the (111) plane of Bi_2_O_3_. Furthermore, the representative energy‐dispersive X‐ray (EDX) mapping imaging of BOBE@BO‐4 presented in Figure 1e illustrates that Bi, O, Br, and Er elements were evenly distributed on the heterostructure surface. These results demonstrate that the BO nanosheets made tight interfacial contact with the BOBE surface through the in‐situ growth process, resulting in the successful formation of BOBE@BO heterostructures.

The observed core–shell structure not only ensures an intimate and large interfacial contact, but also substantially increases the specific surface area and concentration of active sites, which further contributes greatly toward improving catalytic performance.^[^ ^32^ ^]^ This supposition can be validated according to the BET specific surface areas obtained for the samples. As shown in Figure S4a and Table S1 (Supporting Information), BOBE@BO‐4 exhibited the highest BET specific surface area of 32.03 m^2^∙g^−1^, suggesting that the core–shell structure contributed to an increased specific surface area. However, unfavorable photocatalytic reactions can be expected when the BOBE surface is entirely coated, as it is in BOBE@BO‐4, because these hampers contact between the BOBE surface and the external environment.

The detailed crystalline structures of the as‐synthesized pure BO, BOBE@BO, and pristine BOBE samples were analyzed by X‐ray diffraction (XRD), and the results are presented in Figure 1f. As can be seen, the XRD pattern of pristine BOBE exhibits characteristic diffraction peaks at 23.97°, 29.06°, and 31.39° corresponding to (112), (114) and (020) crystal planes, and can be assigned to the orthorhombic phase of Bi_3_O_4_Br (JCPDS No. 84–0793). The characteristic diffraction peaks of pure BO are observed at 27.94°, 32.38°, and 46.44°, and exactly correspond to the (111), (200), and (220) planes in the cubic phase of β‐Bi_2_O_3_ (JCPDS No. 27–0052). The XRD patterns of the BOBE@BO heterostructures clearly consist exclusively of diffraction peaks characteristic of the individual Bi_3_O_4_Br and Bi_2_O_3_ phases without other impurity peaks. It is noteworthy that the intensity of peaks characteristic of the Bi_3_O_4_Br phase exhibit a decreasing trend with increasing BO content, while the intensity of peaks characteristic of the Bi_2_O_3_ phase gradually increases. These results further demonstrate the successful construction of BOBE@BO heterostructures.

The bond chemistry of the samples can be characterized based on the results of Fourier transform infrared (FT‐IR) spectroscopy presented in Figure 1g. Here, the absorption bands located at wavenumbers of 529 cm^−1^ and 1393 cm^−1^ can be attributed to the vibrational modes of single Bi─O bonds,^[^ ^2^, ^21^ ^]^ while the absorption band at 847 cm^−1^ can be attributed to the stretching vibrations of Bi─O─Bi bonds.^[^ ^33^ ^]^ Accordingly, the vibrational peak intensity of the Bi─O─Bi bonds increases with increasing BO loading, indicating that Bi─O─Bi contacts have been constructed between BOBE and BO, and favorable interactions between BOBE and BO have been facilitated by the [Bi─O] tetrahedral sharing.

The surface element compositions and corresponding chemical states of the BOBE@BO heterostructures were evaluated based on the results of X‐ray photoelectron spectroscopy (XPS). The XPS survey spectra of the BOBE@BO heterostructures (Figure S5a, Supporting Information) demonstrate the presence of Bi, O, Br, and Er, which is in line with the previously discussed EDX results (Figure S4b, Supporting Information). The high‐resolution Bi 4f spectra obtained for pure BO, BOBE@BO‐3, and pristine BOBE samples are presented in Figure 1h along with the results of deconvolution based on Gaussian curves. The high‐resolution Bi 4f spectra obtained for pristine BOBE consisted of two strong peaks associated with the spin‐orbit components Bi 4f_7/2_ and Bi 4f_5/2_ of Bi^3+^ at average binding energies 159.52 and 164.81 eV, respectively.^[^ ^30^ ^]^ These same peaks are observed at average binding energies of 159.01 and 164.30 eV for pure BO,^[^ ^33^ ^]^ and at average binding energies of 159.42 and 164.75 eV for the BOBE@BO‐3 sample. Notably, tiny peaks associated with low‐charge Bi ions (Bi^(3‐^ *^x^* ^)+^) were found in BO at binding energies averaging 158.35 and 163.73 eV, suggesting the presence of OVs.^[^ ^34^ ^]^These same minor peaks are observable at average binding energies of 158.41 and 163.93 eV for the BOBE@BO‐3 sample. In terms of the high‐resolution Br 3d spectra obtained for samples presented in Figure S5b (Supporting Information), two fitted peaks associated with the spin‐orbit components Br 3d_5/2_ and Br 3d_3/2_ were observed for BOBE at average binding energies of 68.79 and 69.89 eV, respectively.^[^ ^16^ ^]^ Based on these results, we note that the growth of BO in situ on the BOBE surfaces to form the BOBE@BO heterostructures generated shifts in the fitted Bi 4f peaks associated with the Bi^3+^ and Bi─O environments, and the fitted Br 3d peaks of BOBE@BO‐3 toward lower binding energies compared to those of BOBE, whereas the fitted Bi 4f peaks associated with the Bi^3+^, Bi─O, and Bi^(3−^ *^x^* ^)+^ environments shifted to higher binding energies compared to those of BO. The above results firmly demonstrate the transfer of electrons from BO to BOBE in the BOBE@BO heterojunction owing to the intimate interfacial contact and strong chemical interaction between these components. Such an electron transfer can create an IEF at interfaces in the direction from BO to BOBE, and simultaneously lead to the bending of the energy bands at the interfaces, facilitating the formation of the S‐scheme BOBE@BO heterojunction. The high‐resolution O 1s spectra obtained for pure BO, BOBE@BO‐3, and pristine BOBE samples are presented in Figure 1i along with the results of deconvolution based on Gaussian curves. As can be seen, the O 1s spectrum of pristine BOBE can be deconvolved into three peaks located at binding energies of 529.98, 531.44, and 532.62 eV, and can be attributed to lattice oxygen atoms associated with single Bi─O bonds in the [Bi_2_O_2_]^2+^ layer, oxygen environments including OVs, and oxygen in surface hydroxyl groups, respectively.^[^ ^23^ ^]^ Notably, the relative area under the peak associated with the Bi─O environment in BOBE@BO‐3 was reduced compared to that of the corresponding peak in BOBE, while the relative area under the peak associated with OVs was significantly increased. This can be attributed to the generation of BO in situ, where the original internal Bi─O bond in the [Bi_2_O_2_]^2+^ layer structure of BOBE was broken, and this prompted the generation of OVs. The observed increase in the concentration of OVs in the BOBE@BO heterostructures with increasing BO content is verified by the electron paramagnetic resonance (EPR) spectra presented in Figure 1j.

### 2.2 Optical Absorption Properties

**Figure 2:**
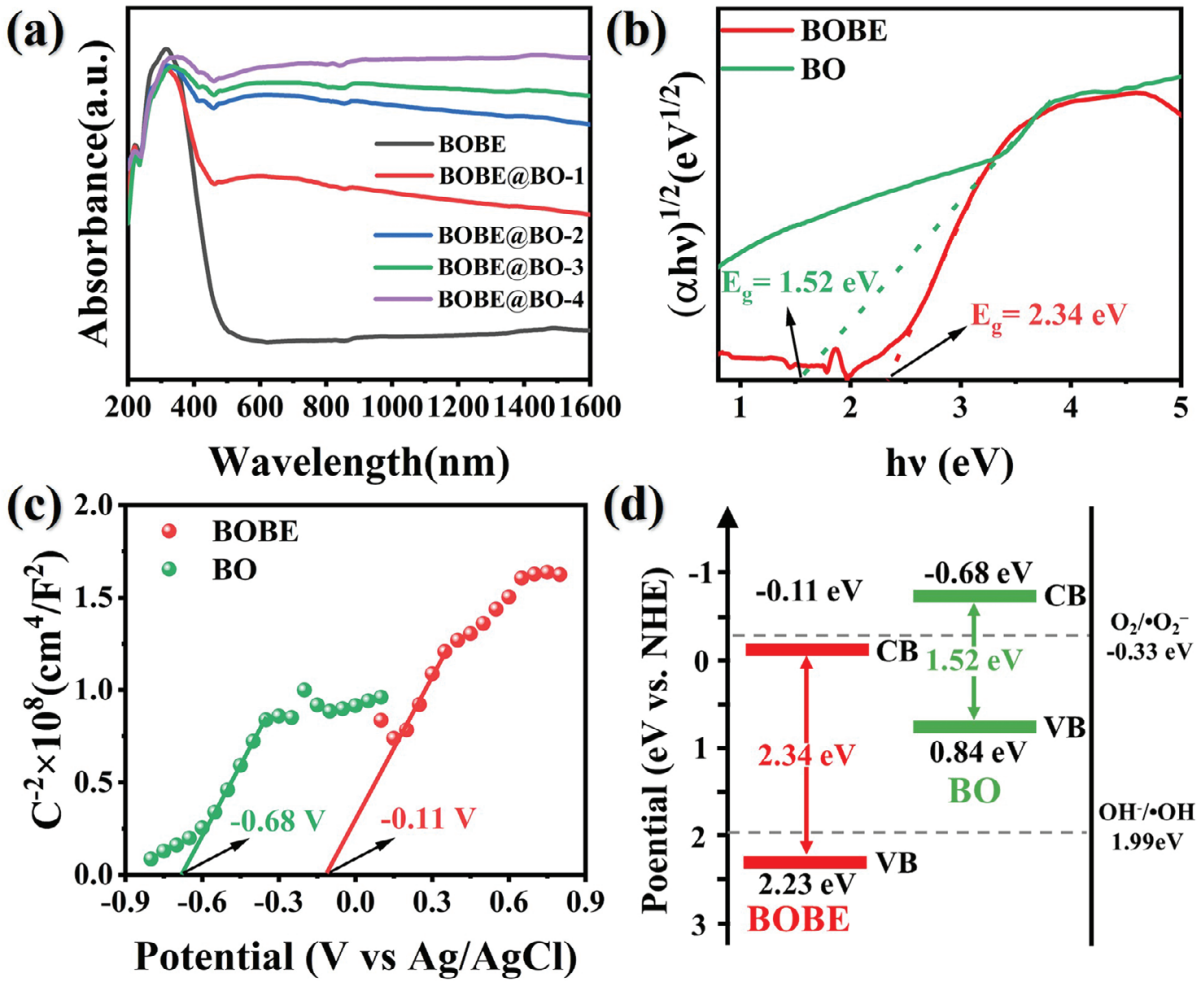
a) UV–Vis‐NIR absorption spectra of BOBE@BO heterojunctions; b) Plots of (αhμ)^1/2^ versus hμ; c) Mott‐Schottky curves and d) Schematic energy band structure of BOBE and BO.

The light harvesting capability of the as‐prepared samples were characterized by UV‐Vis‐NIR absorption spectra. As illustrated in **Figures** **2**a and S6 (Supporting Information), BOBE showed limited Vis light response with the absorption edge at 450 nm, whereas the BO demonstrated light absorption across the Vis to NIR region (400–1600 nm), which can be attributed to the LSPR band.^[^ ^27a^ ^]^ Apparently, the BOBE@BO heterojunction exhibits a wide spectrum of light absorption properties spanning the UV to NIR region. Moreover, the light absorption intensity of the heterojunction was significantly enhanced with increasing BO content, accompanied by the gradual deepening of the sample color from yellow to black (Figure S7, Supporting Information), which implies that the combination of BOBE and BO can enhance solar light utilization ability. The enhanced light absorption ability of the heterojunction will be beneficial for the improvement of the photocatalytic activity.

The UV‐Vis‐NIR absorption spectra of pristine BOBE and BO samples were applied to generate the Tauc‐plots presented in Figure 2b,^[^ ^35^ ^]^ from which the bandgap energies (*E_g_*) of the BOBE and BO samples were calculated to be 2.34 and 1.52 eV, respectively. The energy band structures of the samples can be further characterized according to the Mott‐Schottky (M‐S) diagrams depicted in Figure 2c. Here, the positive slope curves observed in the M‐S plots of BOBE and BO suggest that both materials were typical *n*‐type semiconductors.^[^ ^36^ ^]^ The flat potentials of BOBE and BO versus the Ag/AgCl reference electrode were −0.11 and −0.68 eV, respectively (i.e., 0.09 and −0.48 eV versus the normal hydrogen electrode (NHE)). It is widely recognized that the conduction band (CB) potential of an *n*‐type semiconductor is ≈ 0.2 eV more negative than its flat potential.^[^ ^37^ ^]^ Accordingly, the CB potentials of BOBE and BO can be estimated as −0.11 and −0.68 eV, respectively. The valence band (VB) potentials of BOBE and BO can now be calculated from the CB potentials according to the obtained values of *E_g_* as 2.23 and 0.84 eV, respectively. The obtained energy band structures of BOBE and BO are presented in Figure 2d. Based on the above analysis, the energy band structures of BOBE and BO are arranged in a staggered manner (Figure 2d), and this staggered energy band structure arrangement is the key driver for the construction of S‐scheme heterojunctions.

### 2.3 Photocatalytic Performance Evaluation

#### 2.3.1 Photocatalytic BPA Degradation Performance

To investigate the photocatalytic activity of BOBE@BO heterojunction, BPA was employed as a pollutant for photocatalytic experiments. First, the effect of pollutant auto‐degradation and catalyst adsorption was ensured by blank experiments as shown in Figure S8 (Supporting Information), and furthermore, the adsorption capacity of the catalyst for BPA was evaluated. As displayed in Figure S9a (Supporting Information), the adsorption equilibrium can be achieved after 20 min in the dark. Notably, BOBE@BO‐3 exhibited the highest adsorption performance, which can be attributed to the large specific surface area provided by the core–shell structure.^[^ ^26b^ ^]^ Subsequently, the effect of BPA concentration on the degradation rate was investigated according to the results presented in Figure S9b (Supporting Information). As can be seen, the degradation rate decreased from 80.82% to 43.44% when the BPA concentration increased from 10 to 40 mg∙L^−1^. This decrease in degradation rate can be attributed to the saturation of active sites on the photocatalyst surface at high BPA concentrations.^[^ ^23^ ^]^ Hence, an initial BPA concentration of 20 mg∙L^−1^ was applied in all subsequent degradation experiments.

**Figure 3:**
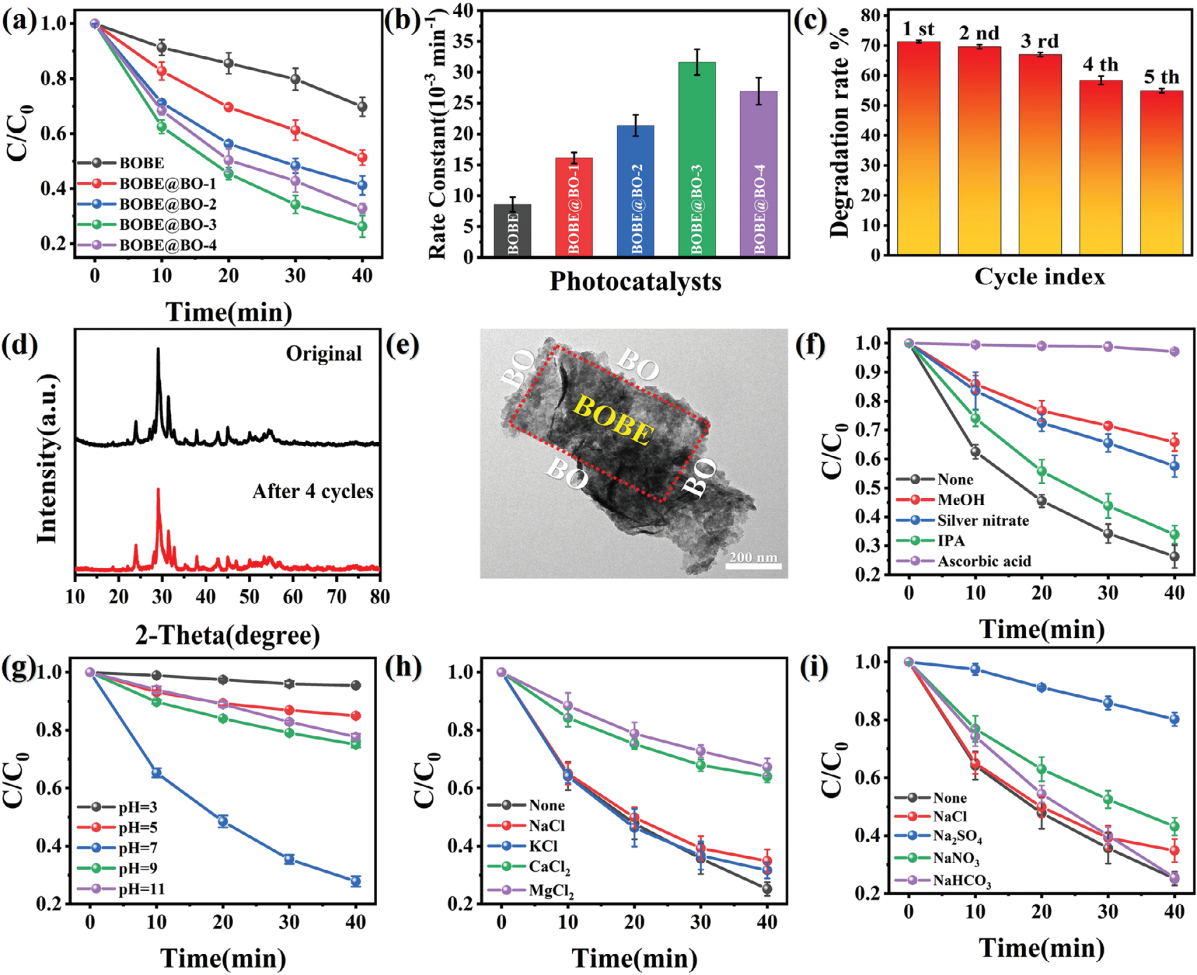
a) Degradation curves of BPA by the BOBE@BO heterojunctions under full‐spectrum irradiation and b) First‐order rate constants; c) Photocatalytic performance cycling experiment of BOBE@BO‐3, d) XRD and e) TEM before and after four reaction cycles; Photocatalytic degradation efficiency of BOBE@BO‐3 in different f) Quenchers, g) pH, h) Cationic and i) Anionic BPA solutions.

The BPA degradation performances of pristine BOBE and the BOBE@BO heterojunction photocatalysts can be evaluated according to the degradation rates observed for the photocatalysts in **Figure** **3**a under full‐spectrum light irradiation. As can be seen, the degradation efficiency of BOBE was only 30.23% after 40 min of full‐spectrum irradiation. In contrast, the photocatalytic efficiency of the BOBE@BO heterojunctions was significantly better, with the highest degradation rate of 72.63% observed after 40 min for BOBE@BO‐3, which was 2.40 times greater than that of BOBE. These results were applied to calculate the reaction kinetic constants (*k*) of the photocatalysts, and the results are plotted in Figure 3b. As can be seen, the values calculated for pristine BOBE, and BOBE@BO‐1, BOBE@BO‐2, BOBE@BO‐3, and BOBE@BO‐4 heterojunctions were 8.58, 16.11, 21.37, 31.64, and 26.95 10^−3^ × min^−1^, respectively. As can be seen, the value of *k* calculated for BOBE@BO‐3 was 3.69 times greater than that of BOBE. The observed decrease in the photocatalytic activity of BOBE@BO‐4 can be attributed to the interference of excess BO on contact between the BOBE cores and the external environment. In addition, other contaminants, such as Cr(VI) and SMX, can also be degraded efficiently (Figure S9c,d, Supporting Information), indicating that BOBE@BO heterojunctions have favorable applicability for different pollutants with various properties. The photocatalytic BPA degradation performances observed for the proposed BOBE@BO heterojunction photocatalysts under full‐spectrum light irradiation are compared with those recently reported for other heterojunction photocatalysts (Table S2, Supporting Information). These results demonstrate that the as‐synthesized BOBE@BO heterojunctions offer significant advantages in the photocatalytic degradation of BPA.

Reusability and stability are of key importance in practical applications. The reusability and stability of the BOBE@BO‐3 photocatalyst were evaluated based on the BPA degradation efficiency obtained over five cyclic degradation experiments conducted under the same conditions. As can be seen from the results presented in Figure 3c, the BPA degradation efficiency of the photocatalyst decreased monotonically from 71.33% to 54.94% after five degradation cycles. This change is partly caused by the unavoidable mass loss of the catalyst over multiple cycles, and partly may be attributed to the decrease in the photocatalytic performance due to the reduction of oxygen vacancies.^[^ ^23^, ^25^ ^]^ In fact, the BOBE@BO‐3 photocatalyst was subject to very little discernible change in crystal structure and morphology before and after cycling. In terms of crystal structure, the XRD patterns shown in Figure 3d exhibit little change before and after four degradation cycles, except for a slight reduction in peak intensity. In terms of morphology, no discernible change can be observed when comparing the TEM image obtained for the BOBE@BO‐3 photocatalyst before degradation cycling (Figure 1c) with a corresponding TEM image presented in Figure 3e obtained after cycling. In terms of chemical states, no discernible change can be observed when comparing the high‐resolution Bi 4f and Br 3d XPS spectra shown in Figure S10a,b (Supporting Information) obtained for the BOBE@BO‐3 photocatalyst after four degradation cycles with those obtained prior to degradation cycling. The O 1s XPS spectra (Figure S10c, Supporting Information) and EPR (Figure S10d, Supporting Information) indicate that the oxygen vacancy content in the material is decreasing, which is due to the fact that during the photocatalytic reaction, the photogenerated electrons can react with the surrounding oxygen molecules or adsorbed oxygen species to fill the oxygen vacancies. This filling effect accumulates as the number of cycles increases, resulting in a gradual decrease in the number of oxygen vacancies, which leads to a decrease in the photocatalytic performance of the material. In conclusion, the BOBE@BO‐3 heterojunctions exhibit not only excellent stability in BPA degradation performance, but also very high stability in terms of chemical structure, making them promising for practical applications.

The BPA degradation process of the BOBE@BO‐3 photocatalyst was evaluated by investigating the active substances involved in the degradation process via free radical quenching experiments conducted under full‐spectrum light irradiation. Specifically, methanol, silver nitrate, isopropyl alcohol (IPA), and ascorbic acid were employed as scavengers for holes (h^+^), electrons (e^−^), hydroxyl radicals (•OH), and superoxide radicals (•O_2_^−^), respectively, and the resulting BPA degradation rates observed for the BOBE@BO‐3 photocatalyst are presented in Figure 3f. As can be seen, the contribution of active species in the BPA degradation process of the photocatalyst decreased in the order of •O_2_^−^ > h^+^ > e^−^ > •OH. While the results indicate that •O_2_^−^ was the main active species by far, the results further demonstrate that h^+^, e^−^, and •OH also participate in the photocatalytic degradation process, although the role of •OH is quite minor. The photocatalytic degradation process of BOBE@BO‐3 for BPA can be further evaluated based on the total organic carbon (TOC) concentration measured in solution during the standard BPA degradation process. The results are presented in Figure S11a (Supporting Information). As can be seen, the TOC mineralization rate of 61.49% was less than the BPA degradation rate of 72.63% observed previously (Figure 3a). Hence, we can conclude that the molecular structure of BPA was effectively cleaved into smaller intermediates during the photocatalytic process.

#### 2.3.2 Effect of Environmental Factors on Photocatalytic Performance

The impact of the initial solution pH on the BPA degradation performance of the BOBE@BO‐3 photocatalyst can be evaluated according to the BPA degradation rates observed for the BOBE@BO‐3 photocatalyst presented in Figure 3g for a variety of pH values under full‐spectrum light irradiation. As can be seen, the best degradation efficiency of 72.19% was achieved at a pH of 7. The unsatisfactory degradation efficiency observed under both over‐acidic and over‐alkaline environments can be attributed to the reaction of •O_2_^−^ with h^+^ to form H_2_O_2_ in the acidic system, and a decreasing concentration of h^+^ under alkaline conditions.^[^ ^38^ ^]^ Furthermore, the pH of the solution can also impact the photocatalytic performance of BOBE@BO‐3 based on its effect on the surface properties of the catalyst and the molecular state of BPA. The mechanism of this effect can be evaluated according to the plot of the zeta potential of BOBE@BO‐3 presented in Figure S11b (Supporting Information) as a function of pH in the range of 3 to 11. These results yield a point of zero charge value for the pH (pH_PZC_) of ≈ 6.79. Accordingly, the photocatalyst surface was positively charged at pH < 6.79 and negatively charged at pH > 6.79. Typically, BPA retains its molecular form at pH values below its dissociation constant (pKa) of 11.2, and is mostly ionized to monovalent or divalent anions after deprotonation at pH >9.^[^ ^11^ ^]^ Based on these results, we can deduce that the BOBE@BO‐3 photocatalyst and BPA molecules may repel each other under extreme acidic or alkaline conditions due to electrostatic repulsion, resulting in lower adsorption of BPA on the active sites of the photocatalyst and poorer photodegradation. Therefore, these results further support the best practice of favoring neutral pH conditions for efficient and sustainable BPA degradation treatment.

In practice, a variety of standard inorganic ions found within wastewater can adversely interfere with the photocatalytic degradation process of BPA. The effect of different inorganic cations (Na^+^, K^+^, Ca^2+^ and Mg^2+^) and anions (Cl^–^, SO_4_^2–^, NO_3_^–^ and HCO_3_^–^) on the degradation efficiency was investigated. As shown in Figure 3h, the degradation efficiency of BPA is only slightly affected by Na^+^ and K^+^ ions, while the inhibition effect of Ca^2+^ and Mg^2+^ is substantial. The latter effect may be because Ca^2+^ and Mg^2+^ ions react with •OH to produce Ca(OH)_2_ and Mg(OH)_2_, which detracts from the degradation of BPA owing to the consumption of •OH.^[^ ^21^ ^]^ In terms of anions, one can see from Figure 3i that Cl^–^ and HCO_3_^–^ have no obvious effect on the BPA degradation activities, while NO_3_^–^ inhibited the catalytic efficiency to some degree. This latter affect may arise because NO_3_^−^ can combine with h^+^, radical •OH and e^−^, resulting in a decreased concentration of radicals. Furthermore, the presence of SO_4_^2−^ strikingly inhibited the degradation of BPA, which can be attributed to declining photocatalytic activity due to the effective scavenging of •OH radicals by SO_4_^2−^.^[^ ^14^ ^]^

#### 2.3.3 Possible Intermediates and Degradation Pathways of BPA

**Figure 4:**
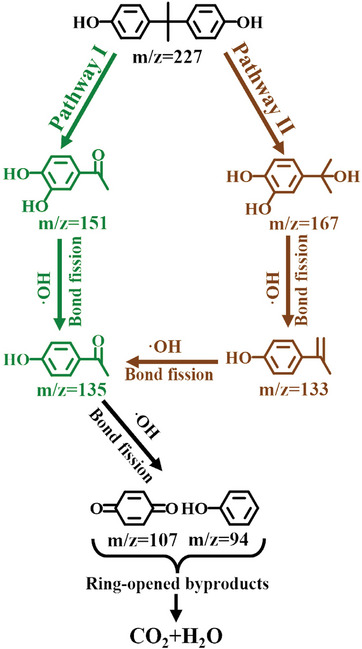
Proposed BPA photodegradation pathways of BOBE@BO‐3 under full‐spectrum irradiation.

The possible degradation pathways of BPA in the BOBE@BO heterojunction photocatalyst system were evaluated based on an exhaustive characterization of intermediates during the photocatalytic degradation of BPA using liquid chromatography‐mass spectrometry (LC‐MS). As shown in Table S3 and Figure S12 (Supporting Information), four intermediates were generated in the presence of h^+^, •OH, and •O_2_^−^ radicals, with products characterized in terms of the mass‐to‐charge ratio (m/z) values of 151, 167, 135, and 133, respectively. Based on these intermediates, the two most probable pathways by which the photocatalytic degradation of BPA proceeds are deduced according to the processes illustrated in **Figure** **4**. The first degradation pathway (Pathway I) involves an electrophilic •OH group attacking the aromatic ring of BPA to form the hydroxylated chemical intermediate 1‐(3,4‐dihydroxyphenyl) ethanone (m/z = 151).^[^ ^39^ ^]^ This intermediate is further dehydrated to form 4′‐hydroxyacetophenone (m/z = 135), which is converted to p‐benzoquinone (m/z = 107) and phenol (m/z = 94) by oxidation. These intermediates are finally degraded to H_2_O and CO_2_. The second degradation pathway (Pathway II) involves the cleavage of BPA to produce the intermediate 4‐(1‐hydroxypropan‐2‐yl) catechol (m/z = 167). Then, the product 4‐isopropenylphenol (m/z = 133) is generated by oxidation and degraded to H_2_O and CO_2_ in the same way as in Pathway I.^[^ ^39^, ^40^ ^]^

### 2.4 Factors Enhancing Photocatalytic Activity

#### 2.4.1 Internal Electric Field Induced by the S‐Scheme Heterojunction

**Figure 5:**
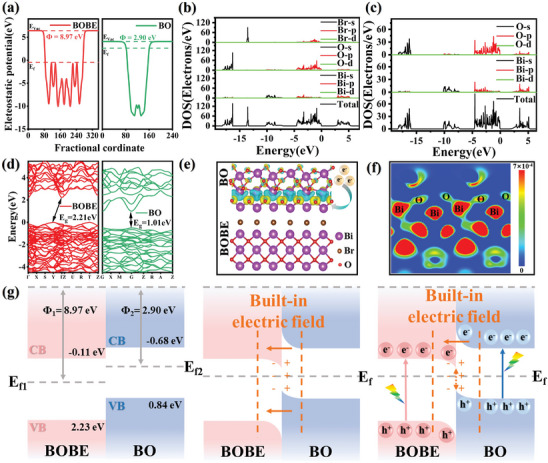
a) Energy band structure, b,c) TDOS and corresponding PDOS, and d) work functions of BOBE and BO; e) 3D charge density and f) planar‐averaged differential charge density of BOBE@BO‐3; g) Schematic of the formation of the IEF.

The mechanism by which the S‐scheme BOBE@BO heterojunctions were formed was evaluated based on the electronic structure properties and the interfacial charge transfer mechanism of the BOBE@BO heterostructure captured via DFT calculations. The band structures and density of states (DOS) of the BOBE@BO heterostructure were investigated based on the structural model presented in Figures S13 and S14 (Supporting Information). The band structures calculated for the BOBE and BO components individually are presented in **Figure** **5**a. As can be seen, these yield an indirect bandgap energy of 2.21 eV for BOBE and direct bandgap energy of 1.01 eV for BO. These values are substantially less than the experimental values obtained for BOBE and BO from the Tauc‐plots presented in Figure 2b (i.e., 2.34 and 1.52 eV, respectively). This deviation between experiment and DFT calculations can be attributed to the tendency of the GGA function in VASP to underestimate the bandgap width of the material.^[^ ^25^ ^]^ The electron DOS of BOBE presented in Figure 5b indicates that the main contributions to the VB of BOBE are the p orbitals of Bi, O, and Br atoms, while the main contributions to the CB are the p orbitals of Bi and O atoms. In contrast, the electron DOS of BO presented in Figure 5c indicates that the main contributions to the VB and CB of BO are the p and s orbitals of Bi and O atoms.

The interfacial charge transfer process in the BOBE@BO heterojunctions can be evaluated according to the work functions (Φ) of the BOBE and BO components obtained via DFT calculations. As illustrated in Figure 5d, BOBE exhibits a much higher Φ value of 8.97 eV than that of BO (2.90 eV), implying that electrons in the BO component can spontaneously transfer to the BOBE component upon contact until the Fermi levels of the two components reach equilibrium. At the point of equilibrium, BO would exhibit an upwardly curved energy level at the interface while BOBE would exhibit a downwardly curved energy level, resulting in the formation of an IEF pointing from BO to BOBE. In accordance with the charge transfer mechanism of an S‐scheme heterojunction, electrons in the VB of BOBE and BO would be rapidly excited to their respective CB levels under light irradiation. Driven by the IEF and bent energy bands, the photogenerated electrons in the CB of BOBE would then spontaneously slide toward BO and recombine with the holes in the VB of BO.

The interfacial charge transfer mechanism of the BOBE@BO heterojunctions can be further investigated based on the 3D charge density illustrated in Figure 5e calculated for the BOBE@BO‐3 structure. As can be seen, the BO surface exhibits a preponderance of blue regions associated with charge depletion at the interface with the BOBE surface. In contrast, the BOBE surface exhibits multiple yellow regions associated with charge accumulation and a relatively limited number of blue regions. These findings demonstrate that negatively charged electrons accumulate on the BOBE surface, while positively charged holes accumulate on the BO surface. This confirms the existence of an IEF pointing from BO to BOBE at the interface of the BOBE@BO heterojunction.^[^ ^29b^ ^]^ Notably, the planar‐averaged differential charge density of the BOBE@BO heterojunction presented in Figure 5f demonstrates the presence of a pathway connected through O─Bi─O bonds at the heterojunction interface for electron transfer from BO to BOB6E.^[^ ^34^ ^]^ The main points of the above analysis associated with the interfacial charge transfer mechanism of the S‐scheme BOBE@BO heterojunction are formally illustrated in Figure 5g, including the unique energy band structure of the BOBE@BO heterostructure, the built in electric field arising upon the equilibration of Fermi levels, and the resulting charge accumulations.

**Figure 6:**
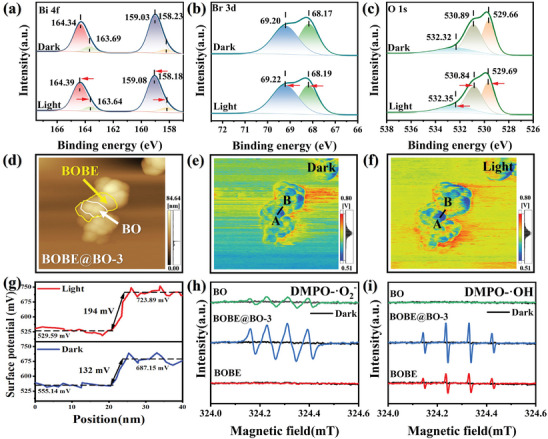
In situ irradiated XPS of BOBE@BO‐3 for a) Bi 4f, b) O 1s, and c) Br 3d; KPFM studies of BOBE@BO‐3: d) AFM images, Surface potential profiles in (e) dark and f) light, g) Potential difference between points A‐B in dark/light; In situ EPR of BOBE, BOBE@BO‐3 and BO for h) DMPO‐•O_2_^−^ and i) DMPO‐•OH.

The above‐discussed charge transfer pathway within the S‐scheme BOBE@BO heterojunction was evaluated experimentally based on an analysis of the high‐resolution Bi 4f, O 1s, and Br 3d XPS spectra obtained for the BOBE‐BO‐3 photocatalyst in situ with and without full‐spectrum light irradiation, which are presented in **Figure** **6**a–c, respectively. As can be seen, the Bi 4f and O 1s peaks associated with BO shifted to lower binding energies after light irradiation, while the Bi 4f and Br 3d peaks associated with BOBE shifted to higher binding energies. The interfacial charge transfer process of the BOBE@BO‐3 photocatalyst can be further assessed according to the direct visualizations based on the AFM images shown in Figure 6d, as well as the corresponding Kelvin probe force microscopy (KPFM) results shown in Figure 6e,f, which were respectively obtained without and with light irradiation. As can be seen from the labeling in the AFM image, points A and B in the KPFM results pertain to BO and BOBE components, respectively. An analysis of the potential difference between points A and B in Figure 6g indicates that the surface potential of BOBE (687.15 mV) was significantly greater than that of BO (555.14 mV) under dark conditions, and the difference was ≈ 132.01 mV. Hence, these results further support the presence of an IEF pointing from BO to BOBE. Under irradiation, the surface potential of BO (529.59 mV) decreased significantly by ≈ 25.85 mV owing to the migration of electrons from the BOBE surface and their accumulation on the BO surface under the IEF, which corresponding increased the surface potential of BOBE (723.89 mV) by ≈ 36.74 mV. Moreover, the S‐scheme charge transfer mechanism was further confirmed by the increasing potential difference of 194.30 mV between the BO and BOBE surfaces.^[^ ^24^, ^30^ ^]^

The efficacy of the S‐scheme charge transfer mechanism of the BOBE@BO heterojunction can be further demonstrated by an analysis of the behavior of photogenerated electrons at the BOBE‐BO interface based on the results of EPR radical trapping experiments conducted for pure BO, BOBE@BO‐3, and pristine BOBE samples using 5,5‐dimethyl‐1‐pyrroline N‐oxide (DMPO) as the capturing agent.^[^ ^11^ ^]^ As shown in Figure 6h,i, the photocatalytic process conducted under full‐spectrum light irradiation was monitored in situ according to DMPO signals involving the superoxide radical (‐O_2_^−^) and hydroxyl radicals (‐OH). The results presented indicate that the radical signal is negligible for all of the samples in the dark. Under light irradiation, the results obtained for BOBE demonstrate that the DMPO‐ •OH signal was clearly observable, while the DMPO‐ •O_2_^−^ signal was practically indiscernible. These features arise because the VB position of BOBE (2.23 eV versus NHE) is greater than the OH^−^/•OH potential (1.99 eV versus NHE).^[^ ^23^ ^]^ In contrast, the results obtained for BO exhibit an observable DMPO‐•O_2_^−^ signal and a negligible DMPO‐•OH signal because the negative CB position of BO (−0.68 versus NHE) is less than the O_2_/•O_2_^–^ potential (−0.33 V versus NHE).^[^ ^41^ ^]^ Importantly, the results obtained for the BOBE@BO‐3 heterojunction clearly exhibit both DMPO‐•OH and DMPO‐•O_2_^−^ signals. Moreover, the intensity of these signals is significantly increased in comparison with those observed for the BOBE and BO samples. These results illustrate that the BOBE@BO‐3 heterojunction photocatalyst possesses both strong oxidation and reduction capabilities for the generation of abundant reactive species, and provide further compelling evidence for the S‐scheme charge separation mechanism.

#### 2.4.2 Heterojunction‐Driven Charge Separation and Transfer

The charge carrier transfer and separation behaviors of the proposed BOBE@BO heterojunctions were evaluated based on a series of experiments conducted for pristine BOBE samples and the different BOBE@BO heterojunction samples. First, the charge transfer resistance was probed by electrochemical impedance spectroscopy (EIS) spectroscopy. For analysis of charge carrier separation, we note that some of the electrons generated during photocatalysis participate in the reduction reaction, while others unfortunately recombine with holes, and thereby generate electromagnetic radiation. Therefore, we evaluated the charge carrier separation efficiency of the samples via an analysis of the photocurrent response obtained under full‐spectrum illumination and the radiative recombination process was captured using photoluminescence (PL) and time‐resolved photoluminescence (TRPL) techniques.

**Figure 7:**
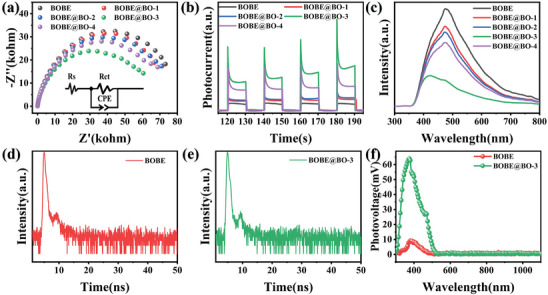
a) EIS, b) Transient photocurrent responses, and c) PL spectra of BOBE@BO heterojunctions; d and e) TR‐PL and f) SPV spectra of BOBE and BOBE@BO‐3.

The EIS results are presented in **Figure** **7**a, along with a diagram given in the inset of the equivalent circuit applied for analysis of the results.^[^ ^22b^ ^]^ Here, the value of interfacial charge transfer impedance (*Rct*) is associated with the radius of the semicircular curve in the impedance spectrum, where the value of *Rct* decreases with decreasing radius. As can see, BOBE@BO‐3 exhibited the smallest semicircle, which suggests that the interface resistance of BOBE decreases after making contact with BO. The values of *Rct* obtained by fitting the EIS curves of all samples to the equivalent circuit are given in Table S4 (Supporting Information). As expected, the *Rct* value obtained for BOBE@BO‐3 (61.04 kΩ) was much lower than that obtained for BOBE (80.96 kΩ). In addition, the enhanced charge carrier transfer of the BOBE@BO heterojunctions can be attributed to the [Bi─O] tetrahedral sharing and the unique core–shell structure, which provide the heterojunctions with larger channels to accelerate the transfer of charge carriers.^[^ ^26a^ ^]^

The photocurrent intensity profiles presented in Figure 7b clearly reflect the efficiencies of the samples for separating the photogenerated charge carriers, where the BOBE@BO‐3 heterojunction exhibited the highest photocurrent intensity of all heterojunctions considered. This can be attributed to the good charge carrier separation of the IEF formed at the BOBE‐BO interface of the heterojunction, which effectively regulates the transfer of photogenerated charge carriers transfer in the heterojunction, and avoids electron‐hole recombination.^[^ ^22b^ ^]^ Similarly, the PL results presented in Figure 7c indicate that the BOBE@BO‐3 heterojunction generated a PL intensity that was much less than those of the other samples, which directly demonstrates that the heterojunction effectively inhibited the recombination of photogenerated charge carriers.^[^ ^32^ ^]^ Additionally, the lifetimes of the photogenerated charge carriers can be evaluated according to TRPL spectra presented in Figure 7d,e for the pristine BOBE and BOBE@BO‐3 samples, respectively. The results obtained from an analysis of the TRPL spectra are presented in Table S5 (Supporting Information). As can be seen, the average charge carrier lifetime (*τ*_ave_) of the BOBE@BO‐3 heterojunction (2.92 ns) was greater than that of BOBE (2.50 ns), which clearly demonstrates that the heterojunction suppressed the recombination of electron‐hole pairs.^[^ ^21^ ^]^ The prolonged carrier lifetime may be attributed to the [Bi─O] tetrahedral sharing S‐scheme heterojunction and core–shell design promoting efficient carrier separation and fast migration, which greatly enhances the utilization of photogenerated carriers in photocatalytic reactions.

Finally, the enhanced charge separation efficiency of the heterojunctions can be further corroborated by the results of surface photovoltage (SPV) spectra presented in Figure 7f for the pristine BOBE and BOBE@BO‐3 samples under full‐spectrum irradiation. As can be seen, both samples exhibit significantly positive SPV signals, suggesting that the photogenerated holes migrated to the sample surfaces under light irradiation, which is a representative property of n‐type semiconductors.^[^ ^41^ ^]^ However, the fact that the intensity of the SPV signal obtained for BOBE@BO‐3 was significantly greater than that of BOBE demonstrates that the sample loaded with BO provided a more efficient charge separation efficiency.^[^ ^3^, ^21^ ^]^ All the above results demonstrate that the construction of the BOBE@BO S‐scheme heterostructure can effectively improve the separation and transport of the photogenerated charge carriers. Therefore, it can be concluded that the BOBE@BO heterojunction has excellent photocatalytic properties.

#### 2.4.3 Utilization of Full‐Spectrum Solar Light

**Figure 8:**
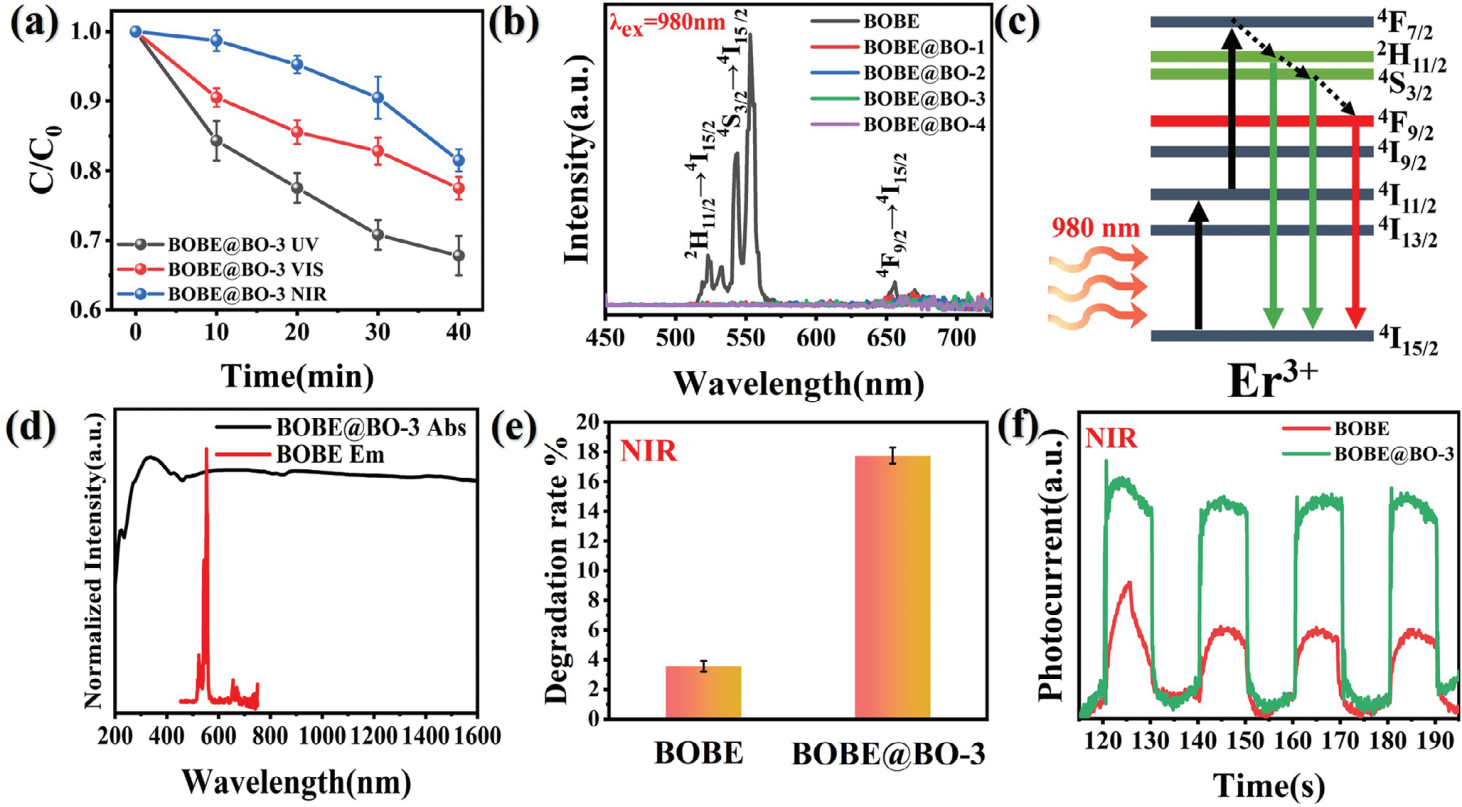
a) Degradation curves spectra of BOBE@BO‐3 to BPA under irradiation at different wavelength bands; b) UCPL spectra of BOBE@BO heterojunctions; c) Energy level diagrams of Er^3+^ ions and possible UC emission processes. d) Overlap of UC luminescence spectra and UV–vis‐NIR absorption spectra of BOBE and BOBE@BO‐3; e) Degradation diagram of BPA and f) Photocurrent response by BOBE and BOBE@BO‐3 in the NIR.

It is known that different spectral regions of incident solar irradiation have different effects on the performance of photocatalytic materials with full spectrum responses.^[^ ^25^ ^]^ In fact, the photocatalytic BPA degradation performance of the BOBE@BO‐3 heterojunction observed strictly under UV, Vis, and NIR light irradiation shown in **Figure** **8**a clearly demonstrates that the BPA degradation efficiency of the heterojunction was much greater under UV (300 ≤ λ ≤ 400 nm) and Vis (420 ≤ λ ≤ 780 nm) irradiation than under NIR (λ > 800 nm) irradiation. Ostensibly, this can be attributed to the direct absorption of high energy UV and Vis irradiation by the photocatalyst.^[^ ^42^ ^]^ This claim is supported by the photocurrent response observed under UV and Vis irradiation shown in Figure S15 (Supporting Information). Nonetheless, the BOBE@BO‐3 heterojunction achieved a BPA degradation efficiency of up to 17.75% in 40 min owing to its enhanced light absorption in the NIR region facilitated by the applied UC engineering strategy.

The efficiency of UC luminescence utilization by the BOBE@BO heterojunctions can be evaluated by analyzing the UC luminescence spectra presented in Figure 8b for pristine BOBE and BOBE@BO heterojunctions under laser excitation at an NIR wavelength of 980 nm. As can be seen, BOBE produced typical UC luminescence emission peaks at Vis wavelengths of 525, 543, and 671 nm under NIR excitation, which can be assigned to ^2^H_11/2_ → ^4^I_15/2_, ^4^S_3/2_ → ^4^I_15/2_, and ^4^F_9/2_ → ^4^I_15/2_ state transitions of Er^3+^ ions,^[^ ^16^ ^]^ respectively, according to the energy levels of these state transitions illustrated in Figure 8c. Notably, no significant UC luminescence can be observed in Figure 8b for the BOBE@BO heterojunctions, which can be attributed to the strong absorption by the heterojunctions of the green and red light emitted through the above‐described UC process.^[^ ^43^ ^]^ The impact of UC luminescence and absorption of the generated Vis light components by the heterojunctions on their full‐spectrum photocatalytic performance can be evaluated further by comparing the BPA photodegradation rates obtained by pristine BOBE and BOBE@BO‐3 photocatalysts after 40 min under NIR irradiation. As can be seen in Figure 8e, the BPA degradation rate obtained by the BOBE@BO‐3 photocatalyst was 4.98 times greater than that obtained by the BOBE photocatalyst. This difference in degradation efficiency can be explained by the fact that more of the low‐energy NIR photons are converted to Vis light through the UC process and are absorbed efficiently in BOBE@BO‐3 than in BOBE (Figure 8d). This claim is further supported by the transient photocurrent response results presented in Figure 8f for the pristine BOBE and BOBE@BO‐3 photocatalysts under NIR irradiation. Accordingly, the above results clearly demonstrate that the UC effect is crucial for enhancing the performance of the photocatalyst in the NIR region of the optical spectrum.

**Figure 9:**
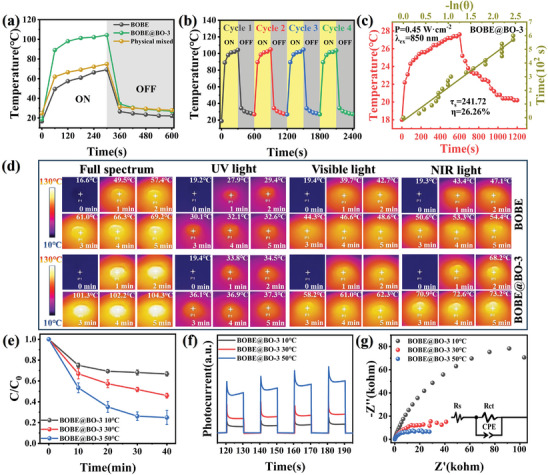
a) Photothermal temperature versus full‐spectrum irradiation time for BOBE, BOBE@BO‐3, and BOBE/BO; b) Photothermal cycling curves of BOBE@BO‐3 under full‐spectrum irradiation (4 cycles); c) The conversion diagram of photothermal efficiency is shown at BOBE@BO‐3; d) Thermal imaging of BOBE and BOBE@BO‐3 under full‐spectrum, UV, Vis and NIR light irradiation; e) Degradation curves of BOBE@BO‐3 to BPA at different temperatures; f) Transient photocurrent response and g) EIS spectra at different temperatures.

Generally, under broad‐spectrum illumination, heat can be produced through a photothermal conversion process, particularly in the near‐infrared region.^[^ ^42^, ^44^ ^]^ Therefore, the photothermal conversion ability of the BOBE@BO heterojunctions was evaluated by comparing the surface temperatures of pristine BOBE and BOBE@BO‐3 samples measured by infrared thermography with respect to irradiation time. In addition to these samples, the surface temperatures of a mechanically mixed BOBE/BO sample were also evaluated for comparison. A representative XRD pattern and SEM image of the BOBE/BO sample are presented in Figure S16a,b (Supporting Information) The measured surface temperatures of the samples with and without NIR irradiation are plotted in **Figure** **9**a Figures S18 and S19 (Supporting Information) as a function of time. As can be seen, the BOBE@BO‐3 heterojunction material exhibited a substantially higher surface temperature under full‐spectrum irradiation than the BOBE or mixed BOBE/BO samples. In fact, the surface temperature of the mixed BOBE/BO sample was not substantially different from that of BOBE sample. This suggests that the unique core–shell structure of the BOBE@BO‐3 material enhances the insulation properties of the material and reduces heat loss.^[^ ^45^ ^]^ In addition, thermal images of the pristine BOBE and BOBE@BO‐3 powder samples obtained at 1 min intervals under different spectral regions of irradiation are presented in Figure 9d. We note that the surface temperature of BOBE@BO‐3 increased rapidly from room temperature to 104.3 °C after 5 min of full‐spectrum illumination, while the corresponding heating rate of BOBE was just 69.2 °C. Hence, the surface temperature of BOBE@BO‐3 was 35.1 °C greater than that of BOBE. For comparison, the results in SI (Figure S17, Supporting Information) indicate that the mixed BOBE/BO sample attained a temperature of only 74.9 °C after 5 min under full‐spectrum irradiation. The enhanced photothermal conversion ability of the BOBE@BO heterojunction can be attributed to its broad‐spectrum absorption and the intense photothermal conversion caused by the LSPR effect of the BO component.^[^ ^27^, ^29^ ^]^ For the other spectral regions, we note that the surface temperature of BOBE@BO‐3 increased to 37.3 °C, 62.3 °C, and 73.2 °C after 5 min under UV, Vis, and NIR irradiation, respectively. It is noteworthy that the surface temperature of BOBE increased to only 54.4 °C after 5 min under NIR irradiation. These results suggest that heat at the surface of the BOBE@BO‐3 heterojunction is mainly generated by incident NIR photons. In addition to the solid‐state photothermal response of the heterojunctions, we also examined the photothermal conversion ability of the BOBE@BO heterojunction powders in photocatalytic BPA solutions (20 mg∙L^−1^) under full‐spectrum irradiation, and the results are presented in Figure S20 (Supporting Information). As was observed in the solid‐phase powder test results, the temperature of the BOBE@BO‐3 solution increased from room temperature to 49.9 °C after 40 min of irradiation, while that of the BOBE solution increased only to 39.5 °C.

The photothermal switching response and photothermal stability of the BOBE@BO‐3 heterojunction can be evaluated based on the surface temperatures recorded in Figure 9b with respect to time over 5 irradiation cycles. The analysis of these results presented in Figure 9c yields a heating and cooling time constant of 241.7 s, and photothermal transfer efficiency of 26.26%. These results can be compared with the corresponding results presented in SI for pristine BOBE (Figure S21, Supporting Information), where the photothermal transfer efficiency of BOBE is observed to be 14.54% less than that of BOBE@BO‐3. The above‐discussed results indicate that the strong photothermal conversion ability of the BOBE@BO heterojunctions can be attributed to three primary factors: 1) the broad spectral response of BOBE@BO enables it to absorb more sunlight; 2) the high‐temperature carriers generated by the LSPR effect of BO lead to a localized temperature increase; 3) the core–shell structure provides insulation to reduce heat loss. As discussed, this strong photothermal conversion ability can be expected to enhance the photocatalytic performance of the heterojunctions by promoting the mobility of photogenerated carriers and the redox reaction rate via the photothermal effect.

#### 2.4.4 Contribution of the Photothermal Effect

The influence of the photothermal effect on the photocatalytic performance of the BOBE@BO heterojunctions was investigated comprehensively by comparing the performance of BOBE@BO‐3 and BOBE for the photocatalytic degradation of BPA under full‐spectrum irradiation with different reaction temperatures controlled by means of a recirculating condensing unit system. As shown in Figure 9e and Figure S22a (Supporting Information), the BPA degradation efficiency of BOBE@BO‐3 and BOBE was significantly enhanced with increasing reaction temperature. Specifically, we note that only 33.25% of the initial BPA concentration was effectively degraded by BOBE@BO‐3 after 40 min of irradiation at a reaction temperature of 10 °C, whereas BPA degradation reached 75.09% at a reaction temperature of 50 °C. The BPA decomposition tests were also conducted under equivalent conditions, but without illumination, to remove the effects of thermocatalytic reactions on BPA degradation, and the results are presented in Figure S22b,c (Supporting Information). As can be seen, none of the samples were effective in catalyzing the decomposition of BPA in the absence of illumination. In fact, the BPA concentration in the solution gradually increased over time as the solvent became increasingly evaporated at a solution temperature of 50 °C.^[^ ^23^ ^]^ Accordingly, the photothermal effect exhibits a significant synergistic impact on the photocatalytic reaction, and this synergistic effect promotes the photocatalytic BPA degradation efficiency rather than contributing to the thermocatalytic reaction.

The impacts of the photothermal effect on the dynamics of photogenerated charge carriers can be evaluated based on the photocurrent response curves and EIS plots respectively presented in Figure 9f,g for BOBE@BO‐3 photocatalysts under full‐spectrum irradiation at different temperature conditions. The results demonstrate that the photocurrent responses of the photocatalysts increased substantially with increasing temperature, while, as can be seen from Table S6 (Supporting Information), the value of *Rct* associated with the radius of the semicircular curve in the impedance spectrum correspondingly decreased from 206.29 to19.46 kΩ when the temperature increased from 10 to 50 °C. Accordingly, we can attribute the enhanced photocurrent response of the photocatalyst to the significant increase in the excitation efficiency of photogenerated charge carriers under high temperature conditions, as well as the reduction of carrier transport resistance, which in turn enhances the charge migration process.^[^ ^45^ ^]^ According to the basic principles of semiconductor physics, the following relationship exists between the temperature (*T*), charge carrier mobility (*μ*), and resistivity (*ρ*) of semiconductors.^[^ ^25^ ^]^

(1) $$\mu \alpha \left[\exp\left(\frac{h\omega }{2\pi k_{0}T}\right)-1\right]$$

(2) $$\rho =\frac{1}{nq\mu_{n}+pq\mu_{p}}$$

here, Equation (1) indicates that the value of *μ* increases with increasing *T*, and includes Planck's constant (*h*), angular frequency (*ω*), and Boltzmann's constant (*k*_0_), while the electron concentration (*n*), hole concentration (*p*), charge (*q*), and values of *μ* applicable to n‐type (*μ*_n_) and p‐type (*μ*_p_) semiconductors are used as the key parameters in Equation (2), revealing that *ρ* increases with increasing *μ*_n_ and *μ*_p_. Taken together, temperature is the core element regulating the dynamics of photogenerated charge carriers during the photocatalytic degradation of BPA, and this regulation is particularly significant under high‐temperature environments.

### 2.5 Photocatalytic Enhancement Mechanism

**Figure 10:**
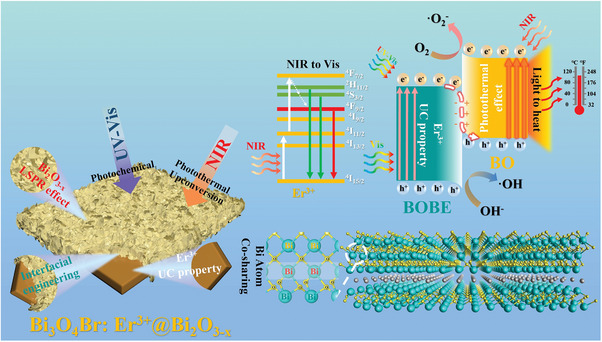
Schematic diagram of the photocatalytic mechanism of BOBE@BO heterojunctions under full‐spectrum irradiation.

Based on the above analysis, the enhanced photocatalytic mechanism of BOBE@BO heterojunction can be inferred, as shown in **Figure** **10**. First, the BOBE and BO components can be excited individually under both UV and Vis irradiation. e^−^ in BOBE transition to the CB, leaving h^+^ in the respective VB. The BOBE component absorbs incident NIR light and converts it to photons in the Vis spectrum via the UC function according to the previously proposed state transition scheme. Under the IEF formed at the interface between the BOBE and BO components, the photogenerated e^−^ on the CB of BOBE combine with the h^+^ on the VB of BO via S‐scheme charge transfer. As a result, e^−^ accumulates in the CB of BO, while h^+^ accumulates in the VB of BOBE. Subsequently, the accumulated e^−^ on BO can react with O_2_ to form •O_2_^–^, while the enriched h^+^ on BOBE can oxidize OH^−^ to produce •OH. Thus, the photocatalytic performance of the BOBE@BO heterojunction is ultimately enhanced by its excellent redox capacity and the accelerated separation of photogenerated charge carriers by the S‐scheme charge transfer mechanism. Second, the LSPR effect of the BO component not only expands the optical response range of the heterostructure from UV to NIR, but also can increases the localized temperature, and thereby accelerates charge carrier transport and chemical reaction kinetics via the photothermal effect. Finally, the interfacial co‐sharing of [Bi─O] tetrahedral further promotes interfacial charge transfer within the heterojunction by providing rapid charge transfer channels, and the core–shell structure promotes the photothermal effect by providing insulation to reduce heat loss.

## 3 Conclusion

The present work addressed the poorly explored implementation of a wide range of schemes for enhancing the photocatalytic performance of bismuth‐based semiconductor materials by integrating UC functionality via the use of an Er^3+^‐doped Bi_3_O_4_Br (BOBE) material with the strong photothermal effect of Bi_2_O_3‐_ *_x_* (BO), and applying the equivalent layer structure of these two materials to facilitate the construction of high‐quality S‐scheme heterojunction interfaces with close atomic‐level contact obtained from the [Bi─O] tetrahedral sharing and the resulting Bi_3_O_4_Br:Er^3+^@Bi_2_O_3‐_ *_x_* core–shell morphology. Combining the UC functionality of Er^3+^ in BOBE with the LSPR effect of BO generated BOBE@BO heterojunctions with broad‐spectrum light absorption capability ranging from the UV to NIR region. Theoretical and experimental analyses demonstrated that the transfer of photogenerated electrons from the BOBE to BO components by the S‐scheme mechanism promotes effective spatial charge separation and preserves the strong reductive and oxidative abilities of photogenerated electrons and holes. Moreover, the interfacial [Bi─O] tetrahedral sharing further promotes interfacial charge transfer within the heterojunction by providing rapid charge transfer channels, and the core–shell structure promotes the photothermal effect by providing insulation to reduce heat loss. As a result, the optimal BOBE@BO‐3 heterojunction exhibited a BPA degradation rate that is 2.40 times and 4.98 times greater than that of BOBE alone under full‐spectrum light irradiation and NIR light irradiation, respectively. Hence, this work demonstrates a new promising strategy for designing high‐efficiency full‐spectrum‐response S‐scheme heterojunction photocatalysts for utilizing solar energy to create renewable and clean energy while degrading pollutants in the environment.

## 4 Experimental Section

All pertinent details can be found in the Supporting Information.

## Conflict of Interest

The authors declare no conflict of interest.

## Supporting information



Supporting Information

## Data Availability

The data that support the findings of this study are available from the corresponding author upon reasonable request.
