# Supplementary material for: Construction of Full‐Spectrum‐Response Bi3O4Br:Er3+@Bi2O3‐ x S‐Scheme Heterojunction With [Bi─O] Tetrahedral Sharing by Integrated Upconversion and Photothermal Effect Toward Optimized Photocatalytic Performance
Source: Adv Sci (Weinh). 2025 Jan 2;12(8):2412214. doi: 10.1002/advs.202412214 (PMC11848554; doi:10.1002/advs.202412214)
Supplement: Supplementary file 1 — Supporting Information [file ADVS-12-2412214-s001.docx]

Supporting Information

**Construction of Full-Spectrum-Response Bi_3_O_4_Br:Er^3+^@Bi_2_O_3-_*_x_* S-Scheme Heterojunction with [Bi-O] Tetrahedral Sharing by Integrated Upconversion and Photothermal Effect Toward Optimized Photocatalytic Performance**

*Zhifeng Li, Liang Xu, Zhaoyi Yin, Junhao Ma, Xiaoyi Dong,* *Shangyong Wang, Zhiguo Song**, Jianbei Qiu, Yongjin Li^*^*

Z. Li, L. Xu, Z. Yin, J. Ma, X. Dong, S. Wang, Z. Song, J. Qiu, Y. Li

Faculty of Materials Science and Engineering, Kunming University of Science and Technology

Kunming 650093, P. R. China

E-mail: liyongjin@kust.edu.cn (Y. Li)

1. **Chemicals and materials**

Bismuth nitrate pentahydrate (Bi(NO_3_)_3_·5H_2_O, AR), Sodium bromide (NaBr, AR), Sodium borohydride (NaBH_4_, 98%), Potassium dichromate standard solution (K_2_Cr_2_O_7_, 0.02 M), Diphenylcarbazide (DPC), Ethanol (AR, 95%), mannitol solution (C_6_H_14_O_6_), Sodium sulfate anhydrous (Na_2_SO_4_, AR, 99%), Nafion solution (Nafion, 5%) were all purchased from Aladdin Reagent. All chemicals were of analytical grade and used as received without further purification.

1. **Preparation of photocatalysts**

Synthesis of Er^3+^ doped Bi_3_O_4_Br nanoplates. Er^3+^ doped Bi_3_O_4_Br nanoplates was prepared by solvothermal method. Totally, 2 mmol of Bi(NO_3_)_3_·5H_2_O, was dissolved in 30 ml mannitol solution (0.1 mol∙L^-1^) to achieve solution A. Totally, 2 mmol NaBr dissolved into 5 ml mannitol solution to obtain solution B. Then add some of Er(NO_3_)_3_ into solution A. Then, solution B was adding into above solution under stirring. After 30 min stirring, now NaOH solution (3 mol∙L^-1^) was employed to tune the pH value to 11.5. Then suspension was sealed in a 50 ml Teflon-lined stainless-steel autoclave, then heat to 160 ℃ for 24 h. After the reaction, the autoclave was cooled down naturally. Then the products were collected and separated by centrifugation, washed with ethanol and deionized water for several times, and then dried at 70 °C in air.

Synthesis of Bi_3_O_4_Br:Er^3+^/Bi. Bi_3_O_4_Br:Er^3+^/Bi were prepared using a solvothermal-calcined tandem synthesis strategy. Weigh 0.5 g of Bi_3_O_4_Br: Er^3+^ and add NaBH_4_ solution with different concentrations (5 mmol∙L^-1^, 10 mmol∙L^-1^, 15 mmol∙L^-1^, 20 mmol∙L^-1^). Subsequently, these mixtures were stirred for 30 min, washed with ethanol and deionized water twice, then dried at 70 ℃ in air, preparation of Bi_3_O_4_Br:Er^3+^/Bi. The sample were named BOBE/Bi-1, BOBE/Bi-2, BOBE/Bi-3, BOBE/Bi-4 according to the concentration of added NaBH_4_ solution.

Synthesis of Bi_3_O_4_Br:Er^3+^@Bi_2_O_3-x_ heterojunctions. Bi_3_O_4_Br:Er^3+^@Bi_2_O_3-_*_x_* heterojunctions were prepared using a solvothermal-calcined tandem synthesis strategy. Weigh 0.5 g of Bi_3_O_4_Br: Er^3+^ and add NaBH_4_ solution with different concentrations (5 mmol∙L^-1^, 10 mmol∙L^-1^, 15 mmol∙L^-1^, 20 mmol∙L^-1^). Subsequently, these mixtures were stirred for 30 min, washed with ethanol and deionized water twice, then dried at 70 ℃ in air, preparation of Bi_3_O_4_Br:Er^3+^/Bi. Weigh 0.2 g of the powder, put it into a 3 ml crucible and oxidize it after annealing at 160 ℃ for 1 h. The sample were named BOBE@BO-1, BOBE@BO-2, BOBE@BO-3, BOBE@BO-4 according to the concentration of added NaBH_4_ solution.

Synthesis of Bi_2_O_3-_*_x_* nanoplates. Weigh 1g commercial bismuth powder into HNO_3_ (0.1 mol∙L^-1^) solution and stir for 30 min. Then the products were collected and separated by centrifugation, washed with ethanol and deionized water for several times, and then dried at 70 °C in air, weigh 0.2 g of the powder, put it into a 3 ml crucible and oxidize it after annealing at 160 ℃ for 1 h.

Synthetic Bi_3_O_4_Br:Er^3+^/Bi_2_O_3-_*_x_* Sample. The synthesised Bi_3_O_4_Br:Er^3+^ and Bi_2_O_3-_*_x_* were mixed according to 1:1 to prepare a physical mixed sample.

1. **Characterization**

The phase structures were observed by the X-ray diffraction measurements using an Ultima IV diffractometer with Cu Kα radiation (λ=1.5406 Å, U=40 kv, I=40 mA). The morphologies and high angle angular dark field-scanning transmission electron microscopy (HAADF-STEM) images of the sample were examined by transmission electron microscopy (TEM, FEI Tecnai G20). For the Infrared (IR) experiments, a Bruker ALPHA FTIR spectrometer equipped was used. The morphology and microstructure were examined by field emission scanning electron microscope (Hitachi Regulus8100, Japan). X-ray photoelectron spectra (Thermo Scientific K-Alpha, America) were tested on a monochromatic Al Kα source and the C(1s) binding energy is 284.8 eV. The absorption edges of samples were tested by a UV-vis spectrophotometer (Hitachi U-4100), with a 150 W xenon lamp as the excitation source, using BaSO_4_ as a reference. Electrochemical properties were characterized by an electrochemical workstation (Gamry, Reference 600+) with a Pt counter electrode, an Ag/AgCl as reference electrode, and 0.5 mol∙L^-1^ Na_2_SO_4_ solution as the electrolyte. The concentration of BPA in aqueous solution was analyzed by UV-vis spectrophotometer (UV-1800, Shimadzu, Japan). The EPR (electron paramagnetic resonance) spectra were recorded on an Electron Paramagnetic Resonance (Bruker A300). The KPFM characterization was performed under ambient conditions using a Bruker Dimension Fastscan (AFM, SPM-9600, Shimadzu).

1. **Photocatalytic degradation experiment**

Catalysts full-spectrum photocatalytic performance for the photocatalytic degradation of BPA contaminants by illumination with a 300 W Xe lamp. Typically, 20 mg photocatalysts was placed in 40 mL BPA solution (20 mg∙L^-1^). The suspension was equilibrated in the dark for a period of time to reach adsorption-desorption equilibrium, then 4 ml suspension was removed every 5 min after sunlight irradiation, and the supernatant was tested after centrifugation. The performance of catalysts for the degradation of bisphenol A was investigated using a 300 W Xe lamp with different wavelength filters. 300 W Xe lamp was used for the photothermal catalysis. The photothermal catalysis experiments were carried out using a 300 W xenon lamp, and the catalytic environment was temperature-regulated using a low-temperature thermostat and water circulation at a distance of 5 cm from the reactor. 20 mg of photocatalyst was added in equal proportions to 40 mL of bisphenol A solution (20 mg∙L^-1^). The suspension was equilibrated in the dark for a period of time to reach adsorption-desorption equilibrium, and then 4 ml of the suspension was removed every 10 min after sunlight exposure. Photocatalytic degradation experiments of SMX using the same approach. The concentration of BPA in aqueous solution was analyzed by UV-vis spectrophotometer at wavelength of 276 nm (SMX= 325 nm). All degradation experiments were repeated three times to eliminate the effects of experimental errors.

The DPC method was used to determine the concentration of Cr(VI) ions in the supernatant solution obtained after photocatalytic experiment. 1 mL Cr(VI) solution obtained after photocatalytic reduction was mixed with 9 mL H_2_SO_4_ (0.2 mol∙L^-1^) aqueous solution in a 20 mL volumetric flask. Then, 0.2 mL of newly prepared 0.25% (w∙v^-1^) DPC in acetone was added to the volumetric flask. After vortexing the mixture about 15 - 30 s, it was allowed to stand for 10 - 15 min to ensure full color development. The red-violet to purple color mixed solution was then measured at 540 nm by the UV-vis spectroscopy using deionized water as reference.

The photocatalytic efficiency was calculated from the following equation (1):

$\eta=\left[ \frac{C_{0}-C}{C_{0}} \right]\times100\%$ (1)

where *η* is the photocatalytic efficiency, *C_0_* is the initial concentration of the contaminants, and *C* is the concentration of the contaminants at the reaction time *t*.

The pH adjustment for adjusting the BPA solution in the subsection on the effect of environmental factors on photocatalytic performance was adjusted by selecting HCl to make the pH of the BPA solution acidic and using NaOH to make the pH of the BPA solution basic. The cations were NaCl, KCl, CaCl_2_, MgCl_2_, and the anions were used as NaCl, Na_2_SO_4_, NaNO_3_, NaHCO_3_, respectively, at an amount of 10 mmol for 1 L of BPA. The bursting agents in the capture experiments were methanol, silver nitrate, isopropanol (IPA), and ascorbic acid. The amounts of methanol and isopropanol were 500 µL for 1 L of BPA, and the amounts of silver nitrate and ascorbic acid were 1 mmol for 1 L of BPA.

1. **Calculation of photothermal conversion efficiency**

To measure the photothermal conversion efficiency of the material, catalysts were added to water to produce a mixture of solutions with a concentration of 0.5 g∙L^-1^. The 4 mL solution was irradiated for 10 min under a xenon lamp (0.45 W∙cm^-1^) with an 850 nm filter, and the temperature change was recorded with a thermal camera. The photothermal conversion efficiency (η) was measured according to the following equation (2 - 4):

$\eta=\frac{\mathrm{hs}\left( T_{\mathrm{Max}}-T_{\mathrm{Surr}} \right)-Q_{\mathrm{Dis}}}{I(1-{10}^{-A})}$ (2)

h is the heat transfer coefficient; s is the surface area of the container. Q_Dis_ represents the heat dissipated. I is the laser power and A is the absorbance at 850 nm.

$hs=\frac{mC_{\mathrm{water}}}{\tau_{s}}$ (3)

m is the mass of the solution containing the photoactive material, C is the specific heat capacity of the solution (C_water_ = 4.2 J∙(g∙℃)^-1^), and τs is the associated time constant.

$t=-\tau_{s}ln(\frac{T-T_{\mathrm{Surr}}}{T_{\mathrm{Max}}-T_{\mathrm{Surr}}})$ (4)

T_Max_ and T_Surr_ are the maximum steady state temperature and the environmental temperature, respectively.

1. **Photoelectrochemical Measurements**

Photoelectrochemical Measurements. The cyclic voltammetry, transient photocurrent response, electrochemical impedance spectroscopy (EIS), and Mott−Schottky curves were carried out on an electrochemical workstation (CHI760E, Shanghai) in a standard three-electrode system with a Pt mesh as the counter electrode, Ag/AgCl (saturated KCl) as the reference electrode, and sample-loaded electrodes as the working electrode in 0.1 mol∙L^-1^ Na_2_SO_4_ aqueous solution (electrolyte solution) at room temperature. The distance between the counter electrode and the working electrode is 2 cm. Indium tin oxide (ITO) with a 1 × 1 cm area photocatalyst was used as the working electrode. The photocurrent measurement of the photocatalyst is measured by several switching cycles of light irradiated by a 300 W xenon lamp (using a 420 nm cut off filter). The photoelectrode in this system is prepared as follows: 10 mg of powder sample was dispersed in 0.2 mL of a mixed solution of ethanol and water (ethanol/water = 1:1 v/v), after adding 5 μL of Nafion solution, the mixture was sonicated for several minutes. And then, the obtained slurry was painted on an ITO glass substrate in an area of 1.0 cm^2^. Mott−Schottky test frequency of 1000Hz.

1. **Theoretical calculations**

The theoretical calculations of band structure were performed based on the density functional theory (DFT) simulations with the Vienna ab initio simulation package (VASP). The core-valence interactions were treated by the projector augmented wave (PAW) method, where the plane wave expansion was truncated with a cutoff energy of 520 eV. Bi_3_O_4_Br:Er^3+^@Bi_2_O_3-_*_x_* used a (3 × 3 × 1) K-point grid for structural optimization in the self-consistent calculations. Based on the calculation results, the valence band maximum and the conduction band minimum lie on the Y and X points, respectively. Hence, this shows the indirect band gap nature. The vacuum space is adopted 15 Å above the surfaces to avoid periodic interactions. The structural optimization was completed for energy and force convergence set at 1.0×10^-5^ eV and 0.03 eV Å^-1^, respectively.

1. **Additional figures**

**
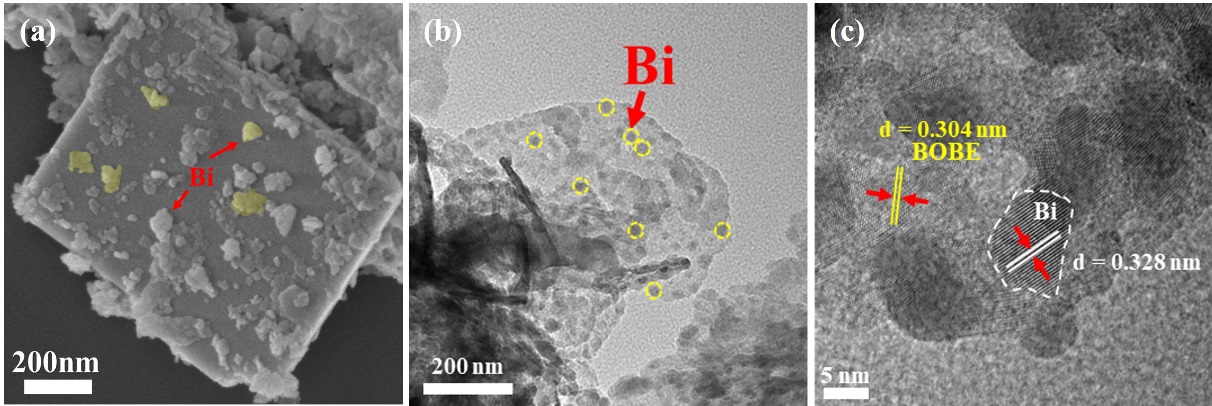
**

**Figure S1.** (a) SEM, (b) TEM, (c) HRTEM of Bi_3_O_4_Br:Er^3+^/Bi-3.

The morphology of the as-prepared BOBE/Bi-3 samples were examined using SEM and TEM. The Bi particles precipitated on the surface of the BOBE sample can be clearly observed from the SEM in Figure S1a, and Figure S1b shows that the TEM further verified the morphology of Bi_3_O_4_Br:Er^3+^/Bi-3 in the SEM. From the HRTEM, two different crystal lattices were observed (Figure S1c). The lattice spacing of *d* = 0.304 nm corresponds to the (114) crystal plane of the Bi_3_O_4_Br. Furthermore, the lattice spacing of *d* = 0.328 nm matched well with the (012) crystal plane of metallic Bi nanoparticles. The HRTEM results indicate that the metallic Bi are directly grown and attached on the Bi_3_O_4_Br:Er^3+^ nanosheets substrate.


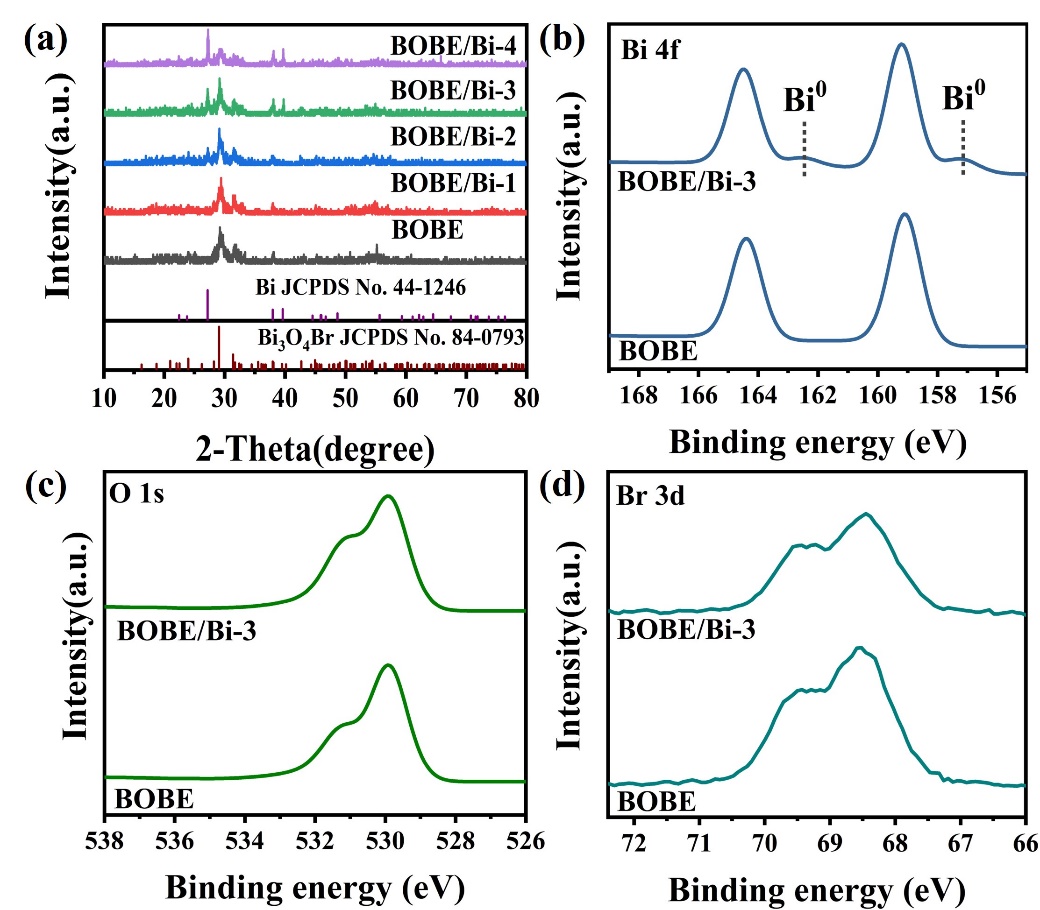


**Figure S2.** (a) XRD, XPS spectra of (b) Bi 4f, (c) O 1s and (d) Br 3d spectra of Bi_3_O_4_Br:Er^3+^/Bi.

The crystalline phases of the synthesized BOBE/Bi samples were determined by XRD analysis. As described in Figure S2a, all the diffraction peaks of the BOBE could be indexed to the orthorhombic phase of Bi_3_O_4_Br (JCPDS No. 84-0793). With the increase of the concentration of NaBH_4_ solution, the positions of the diffraction peaks of Bi_3_O_4_Br almost remained unchanged, which indicates that no phase transformation occurred. Nevertheless, new diffraction peaks appear at 2θ = 27.1° and 39.6°, corresponding to the (012) and (110) crystal planes of Bi-metal (JCPDS No. 85-1331), respectively, identifying the formation of metallic Bi in BOBE/Bi samples. Besides that, the diffraction peaks at 2θ = 27.1° and 39.6° enhance gradually with the increase in concentrations of NaBH_4_ solution, which represents that the Bi content enhanced gradually. This proves that the metal Bi content could be controlled by changing the concentrations of NaBH_4_ solution. The XPS characterization was then performed to further characterize the chemical composition and surface electronic state of the prepared samples. The high-resolution XPS Bi 4f XPS spectra (Figure S2b) show two peaks at 59.1 and 164.4 eV belong to the Bi^3+^ Bi 4f_7/2_ and Bi 4f_5/2_, respectively. Besides, two weak peaks located at 156.7 and 162.0 eV can be assigned to metallic Bi, confirming the presence of metallic Bi on the surface of the BOBE/Bi, which is in agreement with the XRD results. All these characterizations prove the existence of Bi metal in Bi_3_O_4_Br:Er^3+^ after in situ reduction with NaBH_4_.


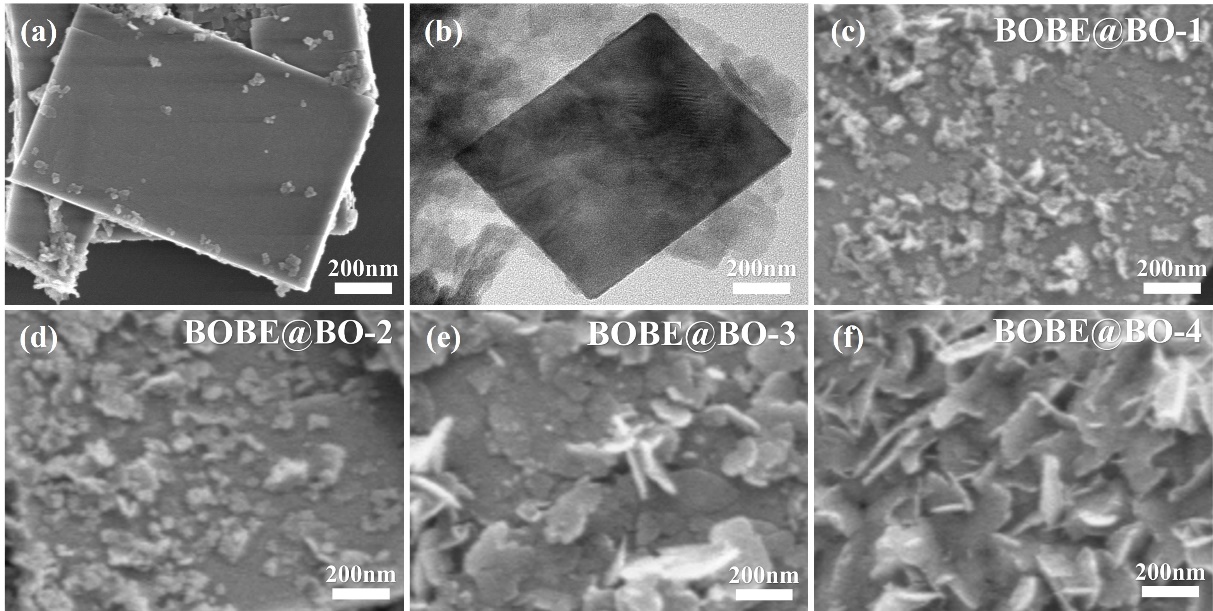


**Figure S3.** (a) SEM and (b) TEM image of BOBE; (c-f) SEM image of BOBE@BO.


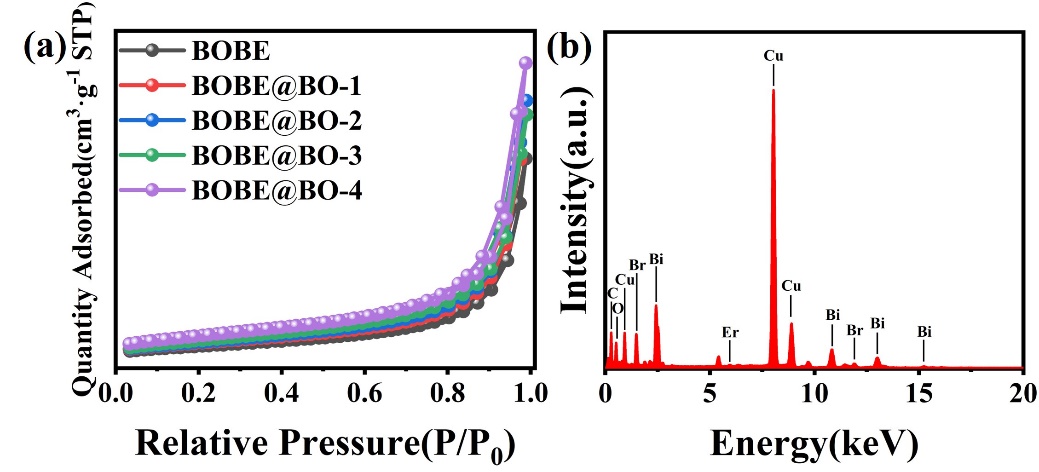


**Figure S4.** (a) BET spectra of BOBE and BOBE@BO; (b) EDS spectra of BOBE@BO-3.

.
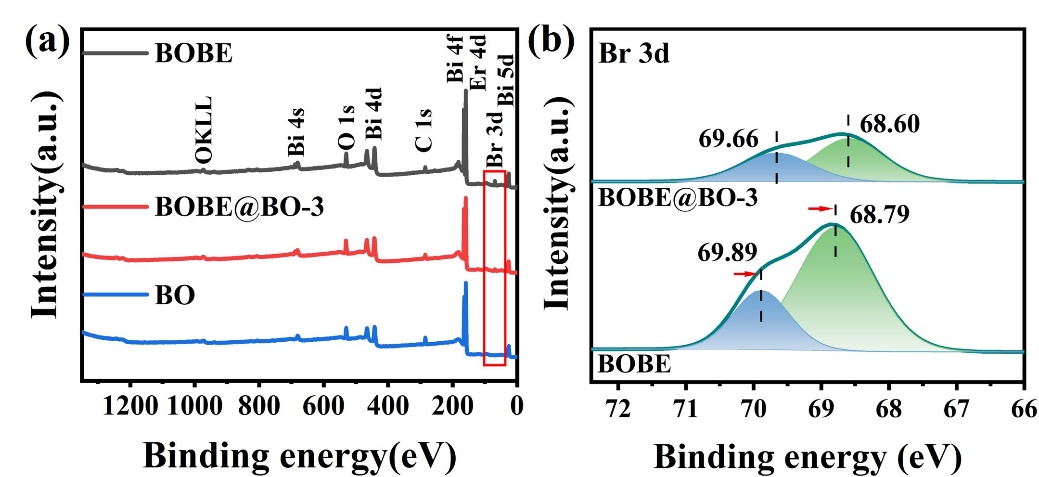


**Figure S5.** (a) XPS image maps and (b)Br 4f spectra of BOBE, BO and BOBE@BO-3.


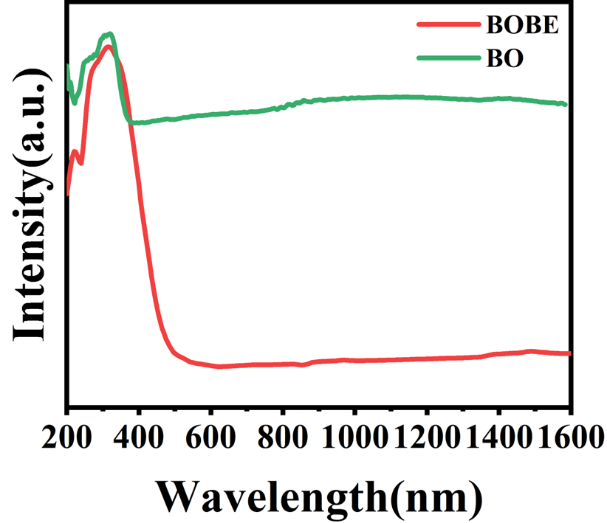


**Figure S6.** (a) UV–vis–NIR absorption spectra of BOBE and BO.


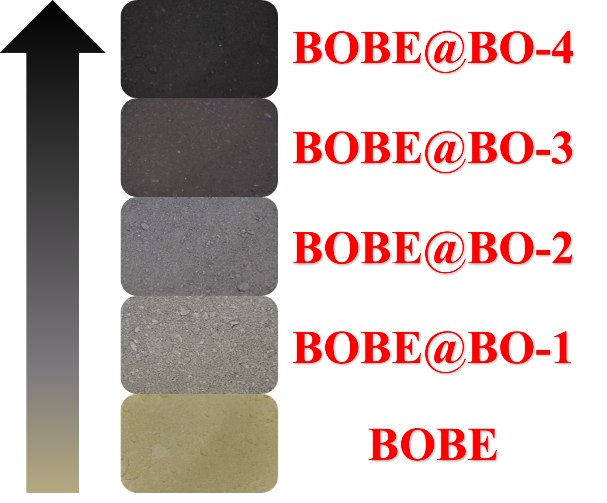


**Figure 7.** Color photographs of heterojunction samples.


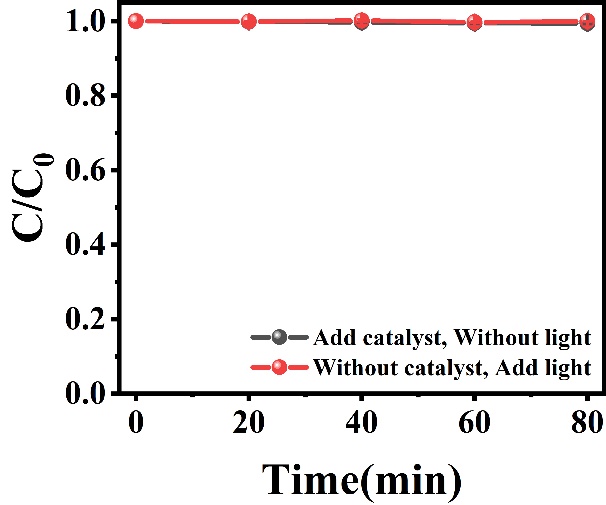


**Figure S8.** Control test of BPA degradation without catalyst or light.


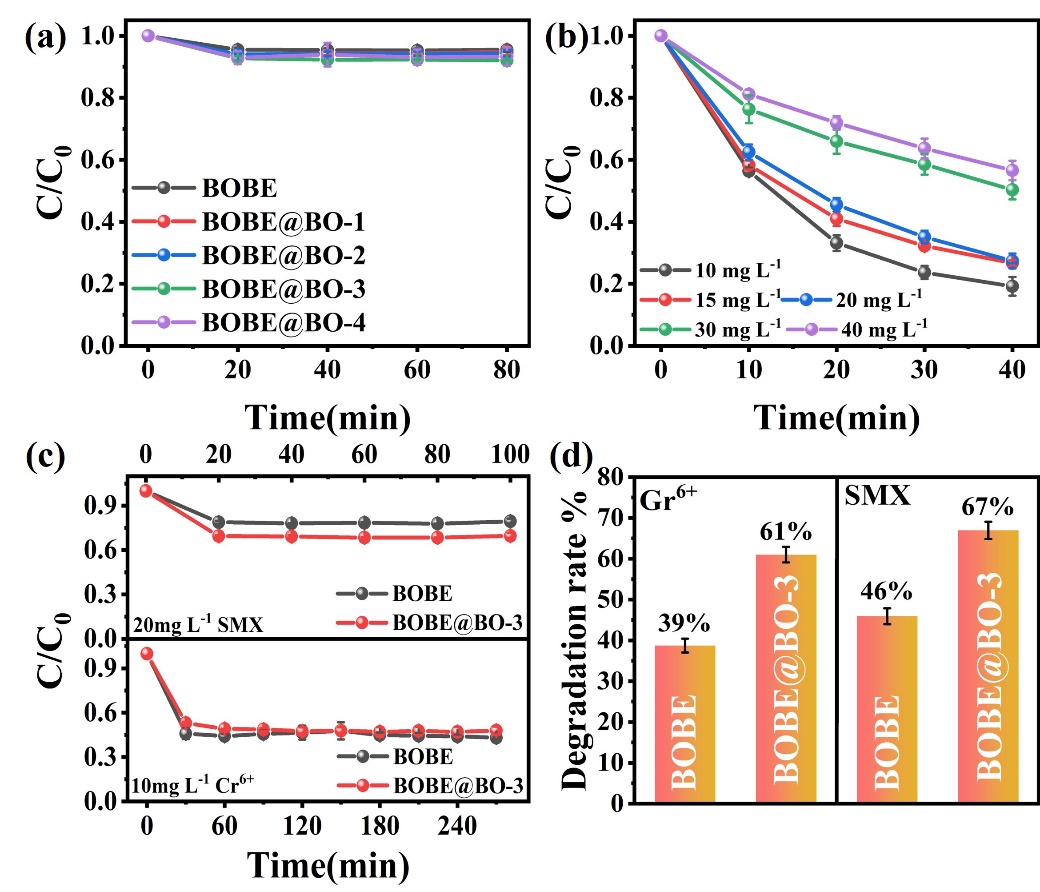


**Figure S9.** (a) Adsorption and desorption curves of BOBE and BOBE-BO for BPA; (b) photocatalytic degradation of BOBE@BO-3 for different concentrations of BPA; and (c) adsorption and desorption curves and (d) plots of degradation rates of BOBE and BOBE@BO-3 for Cr^6+^ (80min) and SMX (40min).


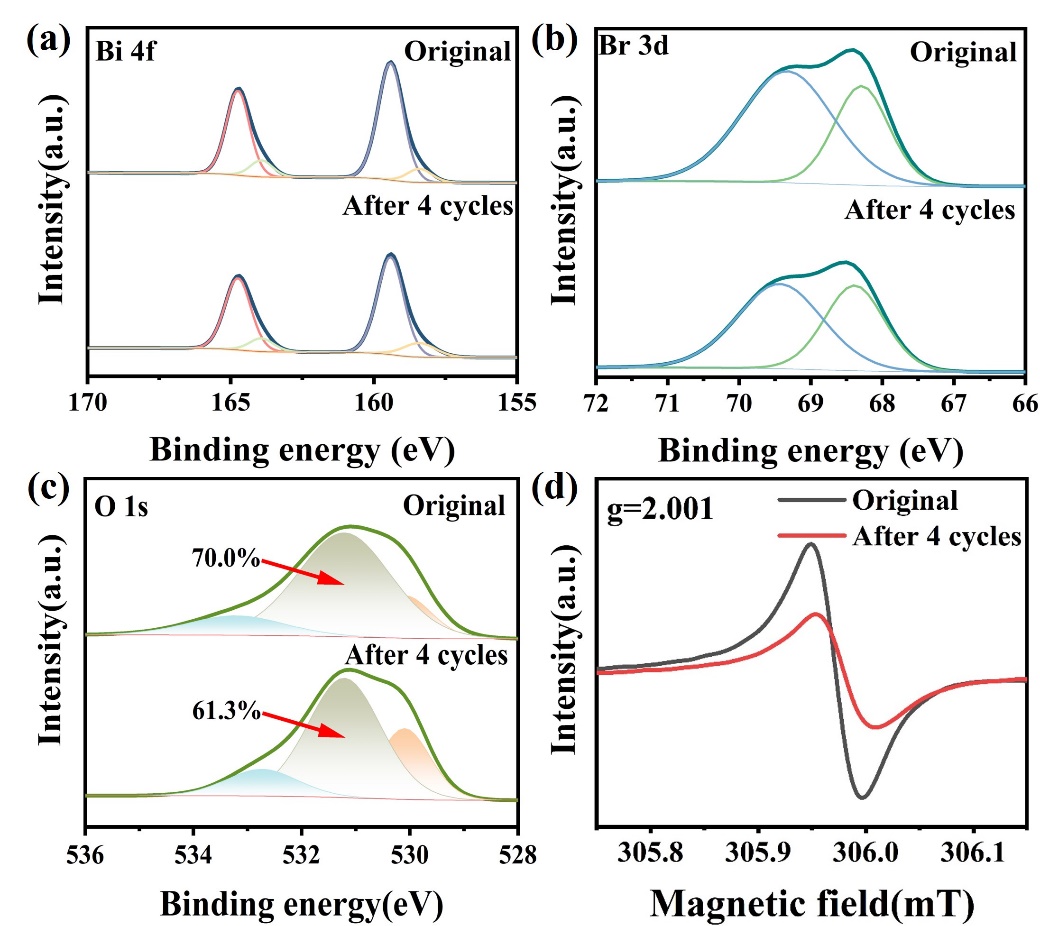


**Figure S10.** XPS spectra of Bi 4f (a), O 1s (b), and Br 3d (c); (d) EPR spectra before and after five reaction cycles.


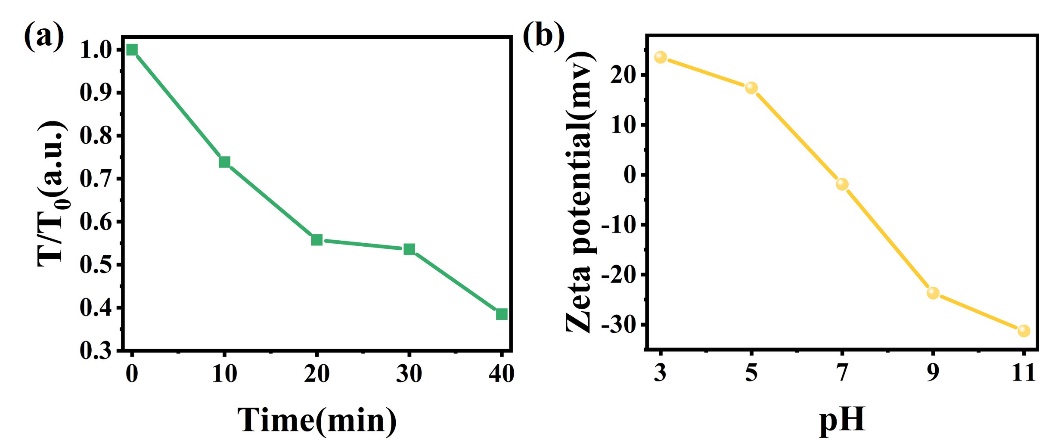


**Figure S11.** (a) TOC performances of BOBE@BO-3; (b) Corresponding zeta potentials table of BOBE@BO-3 under different solution pH values.


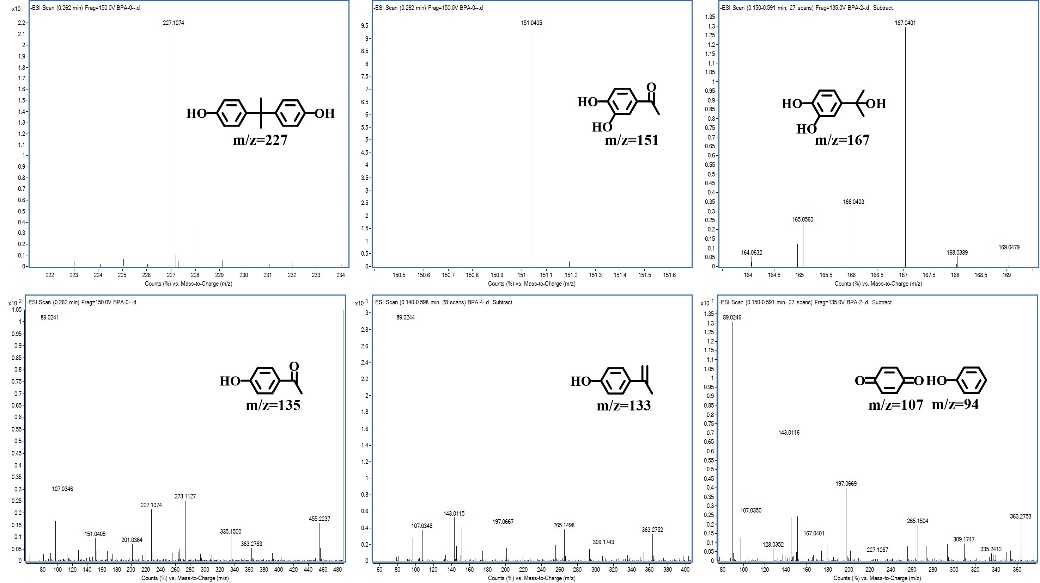


**Figure S12**. MS spectra of the intermediates from BPA degradation over BOBE@BO-3 heterojunction under full-spectrum light irradiation.

**
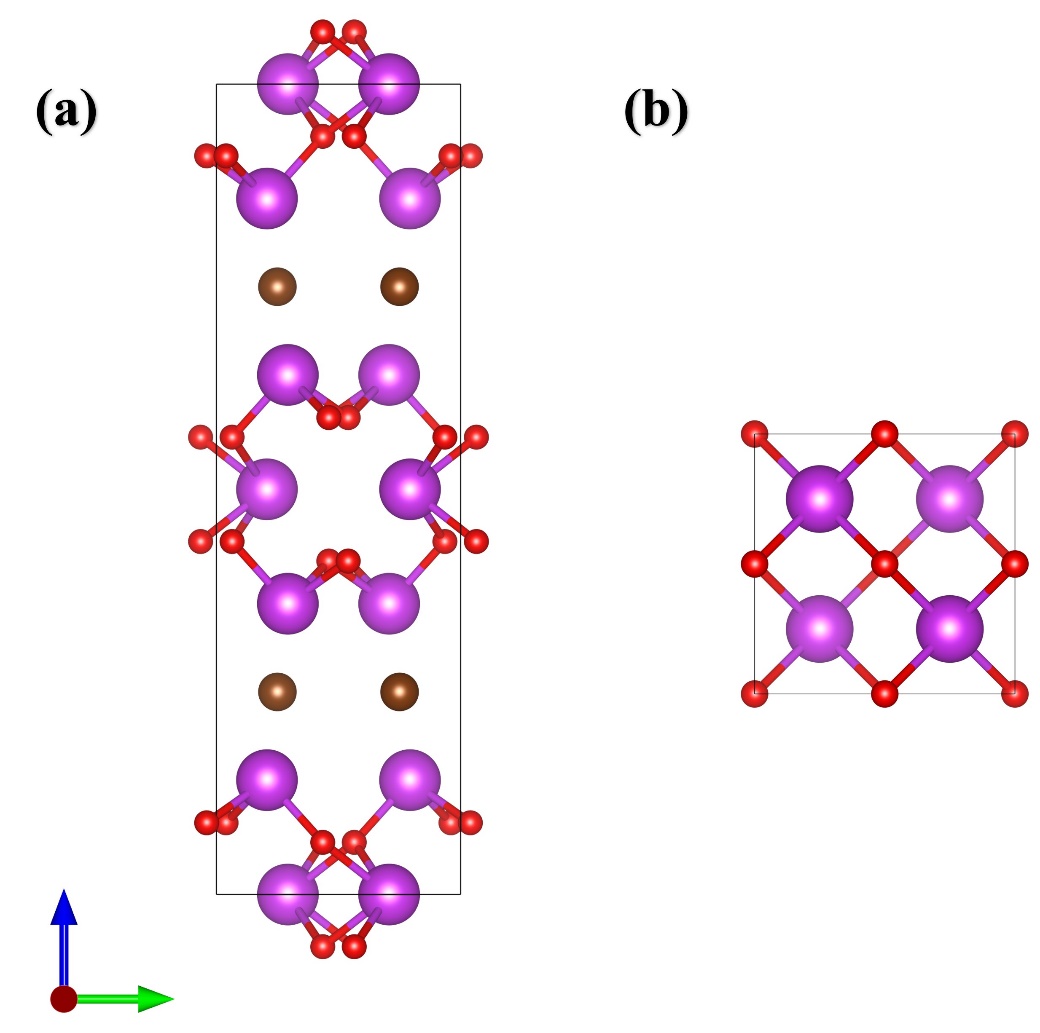
**

**Figure S13.** DFT computational model for (a) BOBE (0 0 1) and (b) BO (0 0 1).


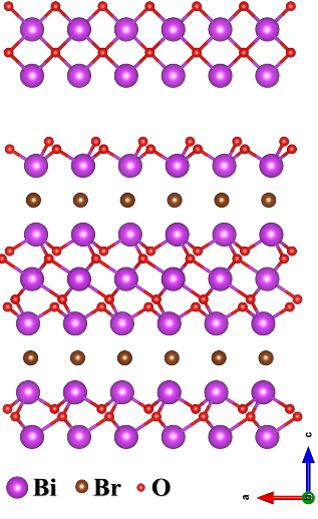


**Figure S14.** DFT computational model for BOBE@BO (0 0 1).


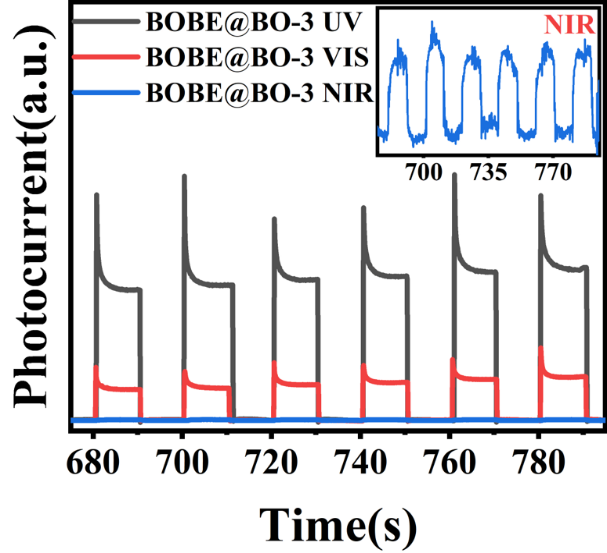


**Figure S15.** Transient photocurrent response spectra under irradiation at different wavelength bands.


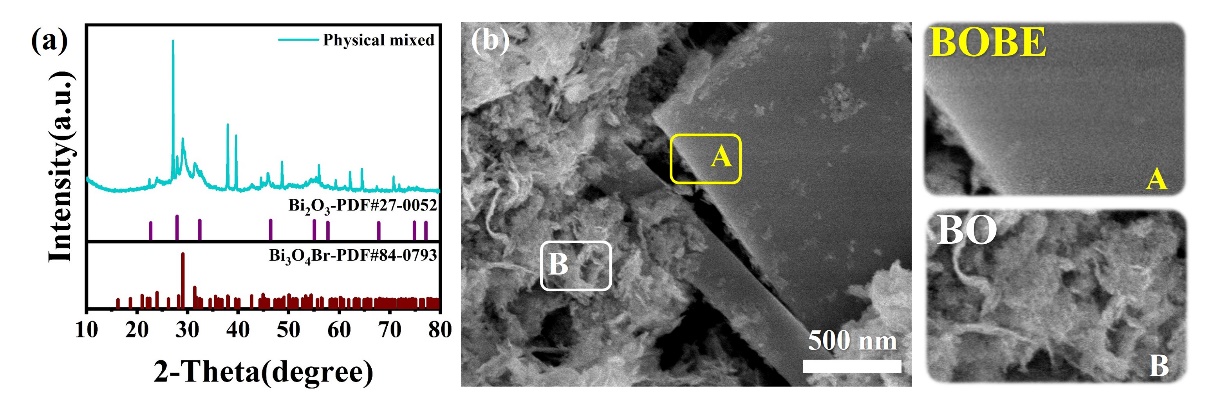


**Figure S16.** (a) XRD and (b) SEM of BOBE and Bi_2_O_3-_*_x_* mechanically mixed samples.


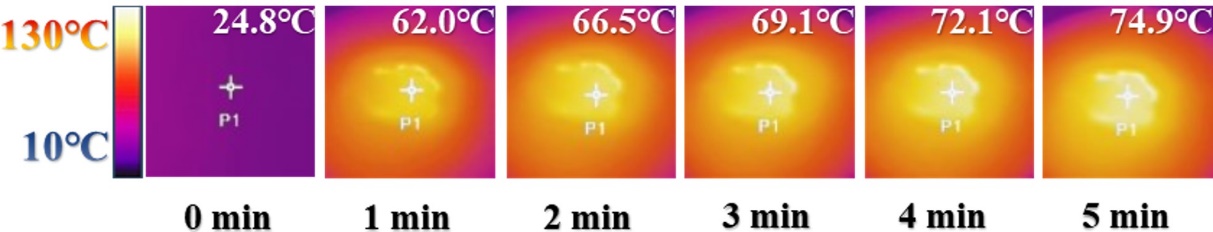


**Figure S17.** Timeline combined with photothermal mapping images for mechanical mixing samples of BOBE and Bi_2_O_3-_*_x_* under full spectrum irradiation.


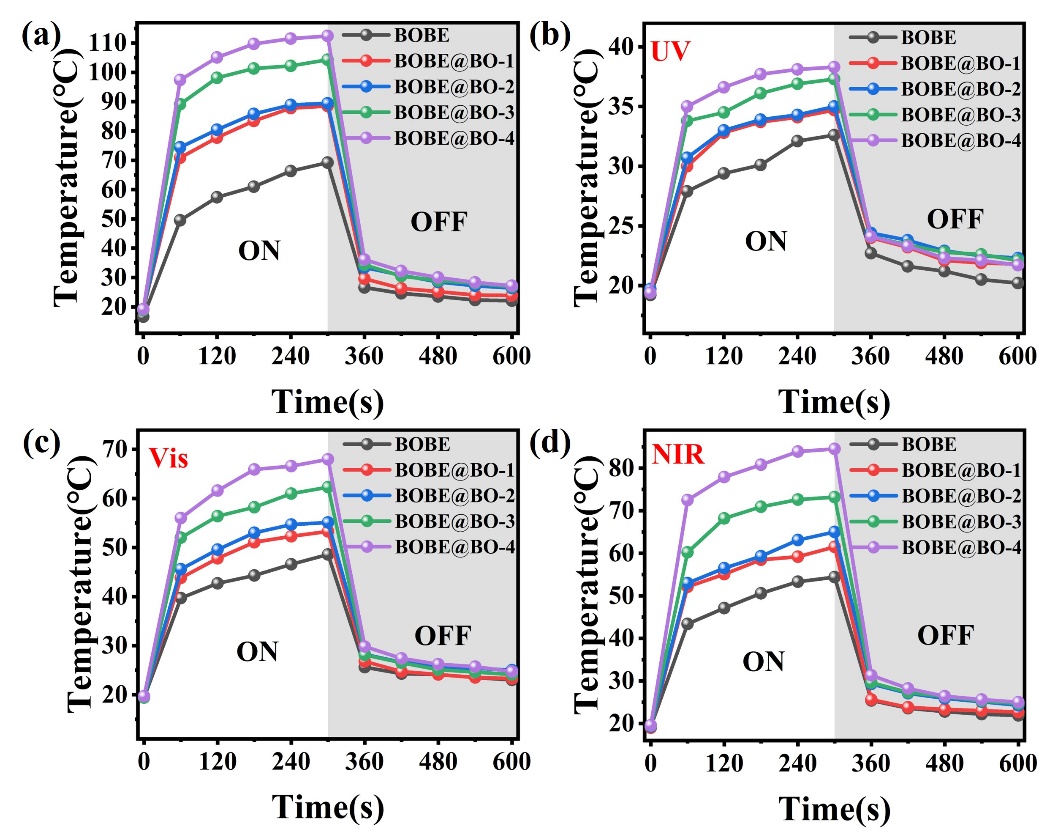


**Figure S18.** Plots of the photothermal temperature changes over irradiation time under full-spectrum and different wavelength bands irradiation.


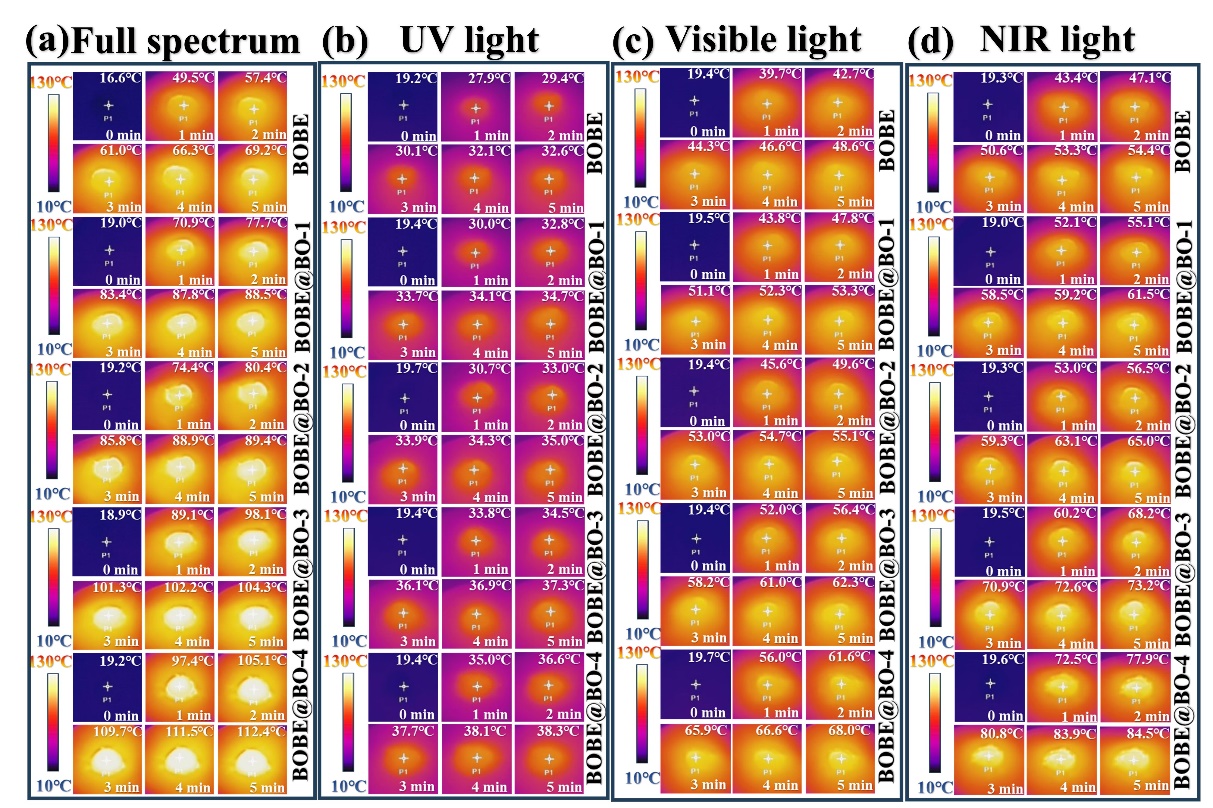


**Figure S19.** Thermal imaging under full-spectrum and different wavelength bands irradiation.


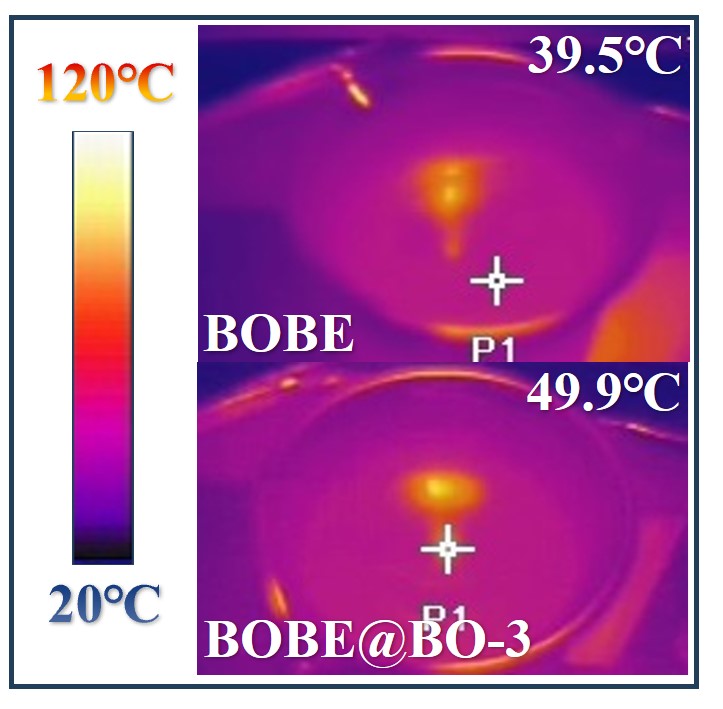


**Figure S20.** Solution temperature diagram of 20 mg BOBE and BOBE@BO-3 catalyst in 40 ml BPA solution under full spectrum irradiation.


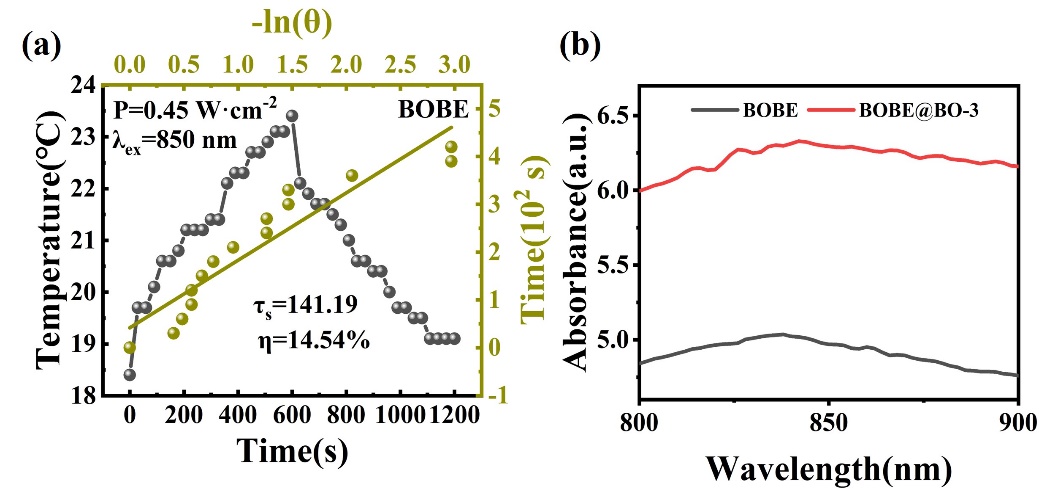


**Figure S21.** (a) Photothermal conversion efficiency (*η*) of BOBE; (b) Absorbance of the prepared sample solution.


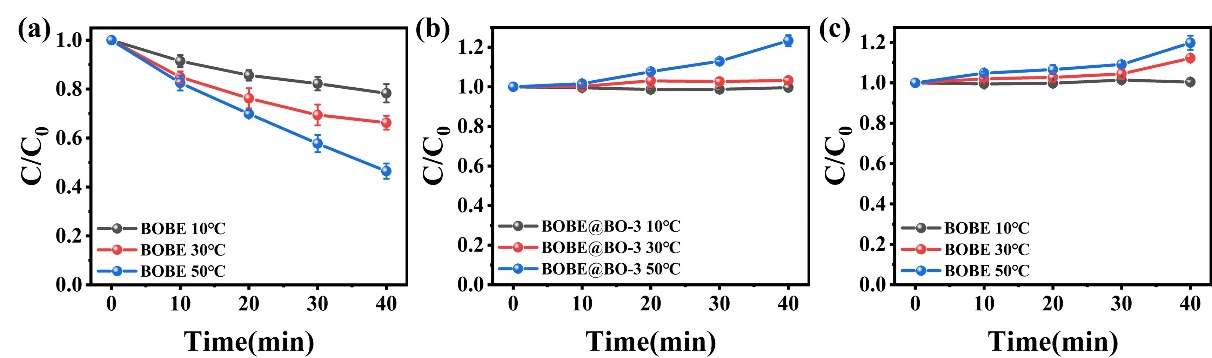


**Figure S22.** (a) Degradation curves of BOBE@BO-3; Thermocatalytic degradation curves of (b) BOBE@BO-3 and (c) BOBE to BPA at different temperatures.

1. **Tables**

**Table S1** BET specific surface area of BO and BOBE@BO.

| Samples | BOBE | BOBE@BO-1 | BOBE@BO-2 | BOBE@BO-3 | BOBE@BO-4 |
| --- | --- | --- | --- | --- | --- |
| BET specific surface area (m^2^ g^-1^) | 19.67 | 23.26 | 24.35 | 26.74 | 32.03 |

**Table S2** Comparison of various photocatalysts for catalytic reduction of BPA.

| Samples | Concentration (mg∙L^-1^) | Dose (g L^-1^) | Light source | | Reaction time (min) | | Removal efficiency (%) | References |
| --- | --- | --- | --- | --- | --- | --- | --- | --- |
| BiOBr/BiVO_4_:Yb^3+^,Er^3+^ | 10 | 0.5 | Full | | | 180 | 89.80 | [1] |
| CoFe_2_O_4_/BiOCl | 10 | 1 | Full | | | 100 | ~40.00 | [2] |
| Bi_4_O_5_Br_2_/α-MnS | 20 | 0.5 | Vis | | | 180 | 78.00 | [3] |
| Bi_2_O_3_/SnO_2_ | 10 | 1 | Full | | | 180 | 93.42 | [4] |
| β-Bi_2_O_3_@BiOI | 20 | 0.06 | Full | | | 60 | 80.00 | [5] |
| LuF_3_:Yb^3+^/Tm^3+^@Lu_6_O_5_F_8_: Yb^3+^/Tm^3+^@BiOI | 10 | 0.5 | | NIR | | 600 | 43.70 | [6] |
| C_3_N_4_@C | 5 | 0.2 | | Vis-NIR | | 300 | 100.00 | [7] |
| Bi_3_O_4_Br:Er^3+^@Bi_2_O_3-_*_x_* | 20 | 0.5 | | Full | | 40 | 70.93% | This work |

DE = degradation effificiency.

**Table S3** Intermediates of BPA identified by LC-MS analysis.

| NO | Chemical name | Chemical formula | structure | m/z |
| --- | --- | --- | --- | --- |
| 1 | Phenol | C_6_H_6_O |  | 94 |
| 2 | p-benzoquinone | C_6_H_4_O_2_ |  | 107 |
| 3 | 4-Isopropenylphenol | C_9_H_10_O |  | 133 |
| 4 | 4’-Hydroxyacetophenone | C_8_H_8_O_2_ |  | 135 |
| 5 | 1-(3,4-Dihydroxyphenyl) ethanone | C_8_H_8_O_3_ |  | 151 |
| 7 | 4-(1-Hydroxypropan-2-yl) catechol | C_9_H_12_O_3_ |  | 167 |
| 8 | BPA | C_15_H_16_O_2_ |  | 227 |

**Table S4** Resistance fitting values of different samples according to the Nyquist polts.

| Samples | Rs (Ω) | Rct (KΩ) | CPE-P (10^-2^) | CPE-T (10^-5^) |
| --- | --- | --- | --- | --- |
| BOBE | 10.14 | 80.96 | 86.91 | 8.56 |
| BOBE@BO-1 | 8.12 | 76.25 | 87.40 | 8.19 |
| BOBE@BO-2 | 7.69 | 74.85 | 87.46 | 8.38 |
| BOBE@BO-3 | 8.29 | 61.04 | 88.10 | 8.94 |
| BOBE@BO-4 | 7.99 | 70.36 | 87.72 | 8.53 |

Rct: charge transfer resistance;

Rs: electrolyte solution resistance;

CPE: constant phase element.

**Table S5** Kinetic parameters for fitting decay parameters of BOBE and BOBE@BO-3 heterojunction.

| Samples | τ_1_ [ns] | A_1_ [%] | τ_2_ [ns] | A_2_ [%] | τ_A_ [ns] |
| --- | --- | --- | --- | --- | --- |
| BOBE | 0.89 | 45.55 | 3.91 | 54.45 | 2.50 |
| BOBE@BO-3 | 0.52 | 37.74 | 4.38 | 62.26 | 2.92 |

**Table S6** Resistance fits of BOBE@BO-3 at different temperatures calculated from Nyquist poles.

| Temperature (℃) | Rs (Ω) | Rct (KΩ) | CPE-P (10^-2^) | CPE-T (10^-5^) |
| --- | --- | --- | --- | --- |
| 10 | 9.70 | 206.29 | 83.28 | 11.28 |
| 30 | 7.32 | 29.81 | 81.57 | 16.70 |
| 50 | 6.78 | 19.46 | 78.55 | 21.55 |

Rct: charge transfer resistance;

Rs: electrolyte solution resistance;

CPE: constant phase element.

**References**

[1] F. Liu, S. Zhang, D. Xu, F. Sun, W. Wang, X. Li, W. Yu, X. Dong, G. Liu, H. Yu, *J. Alloys Compd.* **2022**, 929, 167330.

[2] E. Lin, R. Huang, J. Wu, Z. Kang, K. Ke, N. Qin, D. Bao, *Nano Energy* **2021**, 89, 106403.

[3] F. Chang, S. Zhao, Y. Lei, S. Peng, D.-g. Liu, Y. Kong, *Sep. Purif. Technol.* **2023**, 304, 122324.

[4] L. Chu, J. Zhang, Z. Wu, C. Wang, Y. Sun, S. Dong, J. Sun, *Mater. Charact.* **2020**, 159, 110036.

[5] S. Shu, H. Wang, Y. Li, J. Liu, J. Liu, J. Yao, S. Liu, M. Zhu, L. Huang, *Colloids Surf. B Biointerfaces* **2023**, 221, 112957.

[6] M. Sheng, J. Xue, G. Wu, Y. Liu, Q. Bi, *J. Environ. Chem. Eng.* **2023**, 11, 110784.

[7] Y. Wang, M. Liu, F. Fan, G. Li, J. Duan, Y. Li, G. Jiang, W. Yao, *Appl. Catal. B Environ.* **2022**, 318, 121829.
